# Novel Functionalized Spiro [Indoline-3,5′-pyrroline]-2,2′dione Derivatives: Synthesis, Characterization, Drug-Likeness, ADME, and Anticancer Potential

**DOI:** 10.3390/ijms24087336

**Published:** 2023-04-15

**Authors:** Mohd Asif, Sahir Sultan Alvi, Tazeen Azaz, Abdul Rahman Khan, Bhoopendra Tiwari, Bilal Bin Hafeez, Malik Nasibullah

**Affiliations:** 1 Department of Chemistry, Integral University, Lucknow 226026, Uttar Pradesh, India; machem86@gmail.com (M.A.); arahman@iul.ac.in (A.R.K.); 2 Department of Immunology and Microbiology, South Texas Center of Excellence in Cancer Research, School of Medicine, University of Texas Rio Grande Valley, McAllen, TX 78504, USA; sahir.alvi@utrgv.edu; 3 Department of Biological and Synthetic Chemistry, Centre of Biomedical Research, SGPGIMS-Campus, Raebareli Road, Lucknow 226014, Uttar Pradesh, India; tazeen0@gmail.com (T.A.); btiwari@cbmr.res.in (B.T.)

**Keywords:** isatin-derived spirooxindoles (SOXs), cancer cells, drug-likeness, ADMET studies, ROS generation, and anticancer effects

## Abstract

A highly stereo-selective, one-pot, multicomponent method was chosen to synthesize the novel functionalized 1, 3-cycloaddition spirooxindoles (SOXs) (**4a**–**4h**). Synthesized SOXs were analyzed for their drug-likeness and ADME parameters and screened for their anticancer activity. Our molecular docking analysis revealed that among all derivatives of SOXs (**4a**–**4h**), **4a** has a substantial binding affinity (∆G) −6.65, −6.55, −8.73, and −7.27 Kcal/mol with CD-44, EGFR, AKR1D1, and HER-2, respectively. A functional study demonstrated that SOX **4a** has a substantial impact on human cancer cell phenotypes exhibiting abnormality in cytoplasmic and nuclear architecture as well as granule formation leading to cell death. SOX **4a** treatment robustly induced reactive oxygen species (ROS) generation in cancer cells as observed by enhanced DCFH-DA signals. Overall, our results suggest that SOX (**4a**) targets CD-44, EGFR, AKR1D1, and HER-2 and induces ROS generation in cancer cells. We conclude that SOX (**4a**) could be explored as a potential chemotherapeutic molecule against various cancers in appropriate pre-clinical in vitro and in vivo model systems.

## 1. Introduction

Cancer stands amongst the foremost public health problems worldwide and is the second leading cause of death in the United States. As per the Cancer statistics report of the American Cancer Society, around 2 million new cancer cases and 610,000 cancer-associated deaths have been anticipated to occur in the year 2023 [1]. Prostate cancer is the most prevalent malignancy among males. WHO (2020) reported that 1.41 million new instances of prostate cancer and around 10 million deaths worldwide would be attributed to cancer [2]. Indeed, androgens and androgen receptors play a significant role in the growth and development of prostate cancer. The androgen receptor has been the target of several therapeutic drugs during the past ten years. Moreover, one of the most widely explored membrane-bound receptors involved in cancer cell adhesion, invasion, and metastasis is CD44, which regulates the glycolytic pathway in different carcinomas, hence cell proliferation. The expression of CD44 has been reported to be expressed in prostate-specific PC-3 cells but was absent in LNCaP cells [3,4]. Considering its association with the establishment of prostate cancer, different strategies, i.e., miRNA (miR-383), have been tested for their ability to restrict tumor initiation in CD44-positive prostate cancer cells [5]. 

On the other hand, human epidermal growth factor receptor-2 (HER-2), also known as c-Neu/ErbB2, has shown a profound effect on the pathophysiology of various cancers, most importantly, breast cancer; however, recent studies have already warned that its role in other ranges of cancers cannot be denied [6,7,8,9,10]. Even the genetic defects in HER-2 have also been correlated with the severity of cancers via enhanced HER-2 functionality [7], thereby making it a prime therapeutic target in the management of various cancers. In this context, a set of anti-HER-2 therapeutic regimens have been explored, and among these, the inhibitors of tyrosine kinases are considered the drug of choice for HER-2-positive cancer phenotypes [11]. Like other chemotherapeutic drugs, even the best-studied HER-2 blocker, Lapatinib, has shown serious adverse events, including cardiac toxicity [12].

The steroid 5β-reductase, also termed AKR1D1, plays an integral role in the metabolism and functionality of the 5β-reduced steroids, including androgens and glucocorticoids [13,14]. The interaction of these hormones (androgens) with their receptors, including CD44, is considered the hallmark event for the initiation and establishment of prostate cancer [15,16]. The key strategies for the management of these prostate cancer cases are based on androgen deprivation via targeting AKR1D1 activity [15,17,18]. Finasteride, one of the best characterized and approved AKR1D1 blockers, has widely been prescribed for the initial management of benign prostatic hyperplasia [19]; however, its use has been challenged by distinct adverse events, particularly post-finasteride syndrome, characterized by sexual dysfunction [20].

Another oncogene with a known etiology in different cancers, including prostate, is the epidermal growth factor receptor (EGFR), and the cases having loss of function (LOF) mutations in EGFR showed enhanced efficacy for inhibitors of tyrosine kinase when compared to the cases lacking LOF mutations in EGFR [21,22,23,24]. Moreover, the co-regulation of both HER-2 and EGFR has been reported to fuel the progression and severity of different cancers [25]. On the other hand, an imbalanced redox state, characterized by elevated reactive oxygen species (ROS) generation, has widely been associated with the pathogenesis of various health problems, including ASCVD [26,27], cancer [28], neurodegeneration [29], diabetes [30] and associated complications [31,32]. Therefore, the development of alternative therapeutics against these molecular markers with antioxidant potential is the demand of the time to halt the rising cases of adverse events with commercially available chemotherapeutic as well as hormone deprivation therapies.

**Figure 1:**
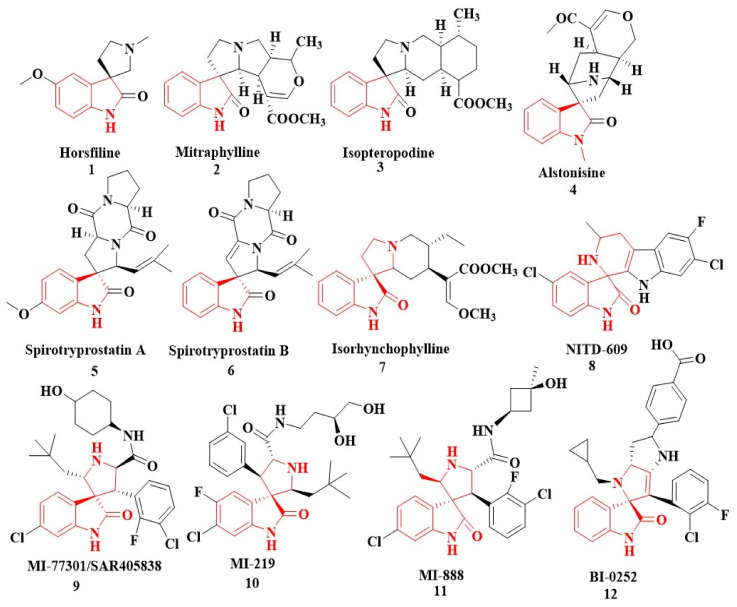
Representation of naturally occurring (**1**–**6**) and synthetic and biologically active SOXs (**7**–**12**).

In nature, different conformations of spirooxindole scaffolds (Figure 1) have been isolated from specific plants, animals, and fungi, which are used for the treatment of various carcinomas [33,34]. Actually, spiro-quaternary-carbon centers have shown greater therapeutic potential in targeting distinct human ailments [35,36,37,38,39]. Therefore, we synthesized spirooxindoles (SOXs) to target prostate cancer. Multicomponent reactions (MCRs) play a significant role in the synthesis of new chemical entities (NCEs) that can be used to develop therapeutic drugs [40]. The MCRs are usually catalyzed in a “single pot reaction” manner in which two or more components react by sequential addition in the same reaction settings. All components in MCRs either assemble in linear form (i.e., Michael addition reaction) or undergo further cyclization (as in 3 + 2 cycloaddition) to give complex molecules, i.e., SOXs [41,42]. Recent studies have widely used the MCRs for the synthesis of novel functionalized SOXs and assessed their therapeutic efficacy against a range of diseases, particularly cancer. Moreover, most of these studies were focused on the assessment of the antiproliferative effect of SOXs against breast cancer, hepatocellular carcinoma, and prostate cancer [41,43,44,45]. Based on the here-mentioned studies, in the current study, we used three different active compounds, namely dioxindoles (DOXs), primary arylamines, and dimethyl acetylenedicarboxylate for the formation of spirooxindole-pyrroline derivatives (**4a**–**h**). Synthesized SOXs (**4a**–**h**) were further subjected to the drug-likeness, ADME, and in silico screening for their inhibitory efficacy against cancer-specific molecular markers, i.e., CD44, AKR1D1, HER-2, and EGFR. Furthermore, the screened SOX (**4a**) was assessed for its antiproliferative impact on PC-3 cells via cytotoxicity, ROS generation, and nuclear condensation analysis.

## 2. Results and Discussion

### 2.1. Chemistry

#### 2.1.1. SOX Synthesis

**Scheme 1:**
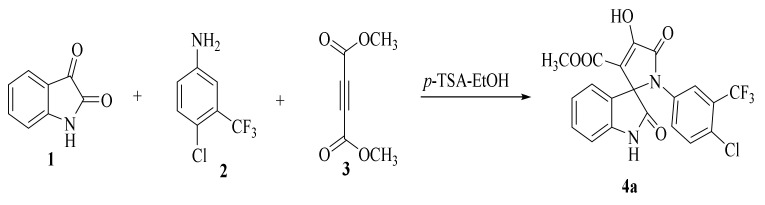
Proposed reaction for the synthesis of the substituted spirooxindole-pyrroline (**4a**).

The synthesis of the target SOXs was optimized using a one-pot, three-component strategy (Isatin derivatives/dioxindoles, arylamines, and dimethyl acetylenedicarboxylates, or DMAD) through a 1, 3-dipolar cyclo-addition reaction in the presence of an acid solution of *p*-toluene sulphonic acid and ethanol (*p*-TSA-EtOH) and purified through column chromatography using n-hexane and ethyl acetate. The proposed reaction (general) for the synthesis of newly formed SOXs is represented in Scheme 1.

Optimization reactions were performed in ethanol at room temperature using 0.0 to 0.8 mM *p*-TSA (*p*-toluenesulphonic acid) as a sole catalyst, wherein 0.5 mM *p*-TSA resulted in the maximum yield (Table 1). 

Compound **4a** was characterized via distinct biophysical approaches (i.e., FT-IR and NMR). Finally, optimized compound **4a** was confirmed through the high-resolution mass spectrometer (HRMS). The remaining SOXs (**4b**–**4h**) were synthesized in the same conditions using various isatins, aryl amine derivatives, and dimethyl acetylenedicarboxylate (Table 2). 

**Scheme 2:**
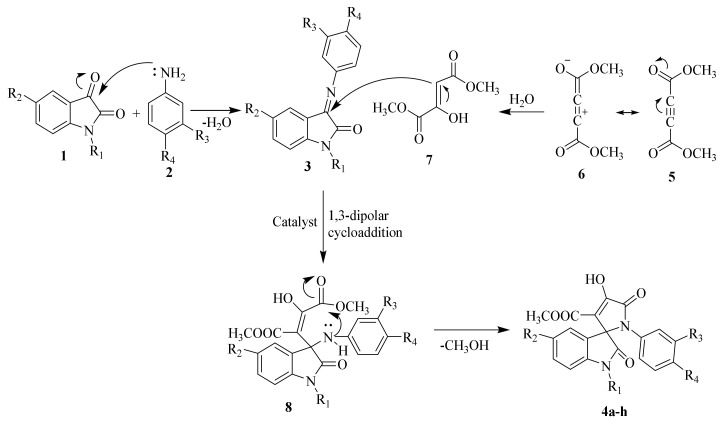
Proposed mechanism for the synthesis of substituted SOXs (**4a**–**h**).

Reaction mechanisms for the synthesis of SOXs have been schematically explained in Scheme 2. To summarize, compounds **1** and **2** reacted at their active sites to form 3-(phenylimino)indolin-2-one (Schiff base) **3**. Simultaneously, H_2_O was nucleophilically added to by the alkyne system of dimethyl acetylenedicarboxylate **6**, followed by a proton shift to generate a 1,3-dipolar intermediate (enamino ester or dimethyl 2-hydroxyfumarate). The Huisgen reaction then occurred between intermediates **3** and **7**, resulting in a 1,3-dipolar cycloaddition product (**8**). To produce stable SOXs, the product (**8**) went through cyclization (**4a**–**h**).

**Scheme 3:**
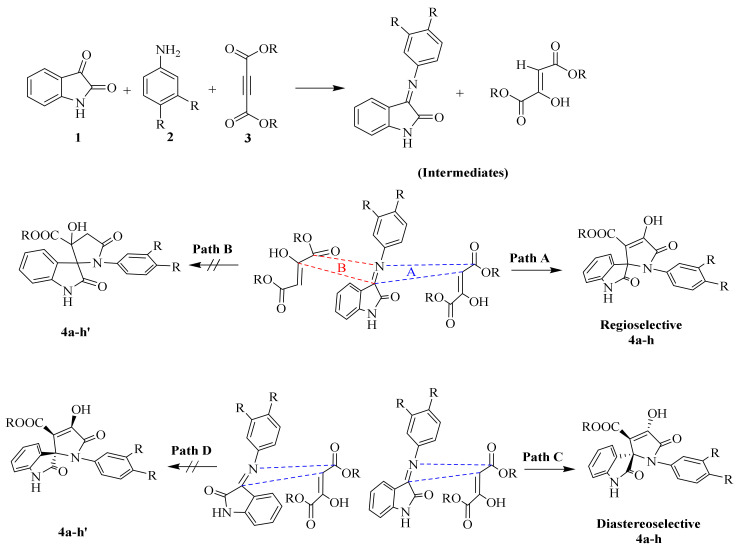
Plausible approach of 1,3-dipolar cycloaddition for the formation of regio- and diastereoselective spirooxindole-pyrrolines (**4a**–**h**).

Further, to predict all the possible interactions between two intermediates (i.e., **3** and **7**) and the possibility of getting desired SOXs, we analyzed the more specific regioselectivity and diastereoselectivity phenomenon (Scheme 3). Taking the regioselectivity into account, the carbonyl group at C-3 of isatin residue of **1** is more active than C-2 of the same due to the possibility of resonance between N-1 and C-2 to form lactim and lactam forms of isatin, making further nucleophilic attacks at C-2 less feasible, whereas, the same phenomenon is not possible at C-3 of intermediate **3**.

On the other hand, the H atom of C-3 of intermediate **7** is active and prone to attack the C-3 of intermediate **3** to form SOXs **4a**–**h**, whereas the C-2 of intermediate **7** contains a -OH group providing it with more electron density and making it unable to generate a carbanion at C-2 required to attack the C-3 of intermediate **3**. Therefore, the reaction proceeds in a Path A-dependent manner, while Path B is aborted. On the other hand, as far as diastereoselectivity is concerned, the newly synthesized SOXs (**4a**–**h**) will attain trans configuration (Path C) rather than cis conformers (Path D; **4a**–**h′**) in order to get more stability as the cis conformers will have -OH and -COOR at same plane making them overcrowded and unstable.

#### 2.1.2. Characterization Details of SOXs (**4a**–**h**)

##### Methyl-1’-(4-chloro-3-(trifluoromethyl)phenyl)-4’-hydroxy-2,5’-dioxo-1’,5’-dihydrospiro[indoline-3,2’-pyrrole]-3’-carboxylate (**4a**) 

Orange solid, 86%, m.p. 243–244 °C; IR-KBr: 3329.34 (OH), 2955 (sp3-CH3), 1715.97 and 1610.97 (C=O), 1461.2 (C-H), 1383.48 (C-H), 1248.04 (C-O), 1153.47 (C-N), 753.71, 599.42 cm^−1^. ^1^H NMR (800 MHz, DMSO-d6) δ = 11.19 (s, 1H, OH), 8.20–8.19 (t, J = 8.1 Hz, ArH), 8.09–8.08 (d, J = 8 Hz, ArH), 7.95–7.93 (m, ArH), 7.89–7.88 (d, J = 8 Hz, ArH), 7.75–7.68 (t, J = 8.2 Hz, ArH), 7.60 (s, ArH), 7.67–7.52 (m, ArH) 7.46–7.44 (m, ArH), 7.10–7.08 (t, J = 8 Hz, ArH) 6.95–6.94 (d, J = 8 Hz, ArH), 3.43 (s, 3H, OCH3) ppm. ^13^C NMR (75.5 MHz, DMSO-d6) δ = 184.80, 168.82, 163.40, 159.23, 146.57, 138.47, 134.72, 132.34, 128.83, 125.97, 123.17, 118.85, 111.69, 53.88, 53.65 ppm. HRMS (ESI-TOF *m/z*) (M + H) + calculated for C_20_H_13_ClF_3_N_2_O_5_+ 454.00784, found 453.046; 454.0439; 455.0456; 456.0508; (M + Na) + calculated for C_20_H_12_ClF_3_N_2_NaO_5_+ 475.9897, found 475.0304 (Please refer to supplementary figures).

##### Methyl-1’-(3-chloro-4-fluorophenyl)-4’-hydroxy-2,5’-dioxo-1’,5’-dihydrospiro[indoline-3,2’-pyrrole]-3’-carboxylate (**4b**)

Yellow solid,79%, m.p. 241–243 °C; IR-KBr: 3324.03 (OH), 2890 (sp3-CH3), 1734 and 1615 (C=O), 1463 (C-H), 1384 (C-H), 1209 (C-O), 1057.54 (C-N), 752.4, 550 cm^−1^. ^1^H NMR (800 MHz, DMSO-d6) δ = 11.022 (s, 1H, OH), 7.610–6.480 (m, 7H, ArH), 3.392 (s, 3H, OCH3) ppm. ^13^C NMR (100 MHz, DMSO-d6) δ = 182.01, 169.12, 65.37, 154.05, 157.11, 156.34, 144.71, 143.02, 139.11, 137.59, 134.01, 131.37, 129.08, 128.04, 121.69, 111.07, 197.77, 72.48, 54.18 ppm. 1H NMR (300 MHz, DMSO-d6) δ = 11.02 (s, 1H, OH), 10.90 (s, 1H, NH), 7.610–6.48 (m, 7H, ArH), 3.80 (s, 3H, OCH_3_) ppm. HRMS (ESI-TOF *m/z*) (M + H) + calculated for C_19_H_13_ClFN_2_O_5_ + 404.00784, found 403.0468; 405.044, (M + Na) + calculated for C_19_H_12_ClFN_2_NaO_5_ + 425.9897, found 425.0281; 427.0275.

##### Methyl-4’-hydroxy-2,5’-dioxo-1’-phenyl-1’,5’-dihydrospiro[indoline-3,2’-pyrrole]-3’-carboxylate (**4c**)

Yellow solid, m.p. 242–245 °C; IR-KBr: 3268 (OH), 2860 (CH3), 1732 and 1614 (C=O), 1463 (C-H, methyl), 1384 (C-H, ester), 1337 (C-N, Aramine), 1207 (C-O, ester), 1073, 751, 694 cm^−1^. ^1^H NMR (800 MHz, DMSO-d6) δ = 11.00 (s, OH), 10.80 (s, NH), 7.58 (dddd, J = 7.3, ArH), 7.49 (m, ArH), 7.47 (m, ArH), 7.30 (m, ArH), 7.05 (m, ArH), 6.99 (m, ArH), 6.91 (m, ArH), 6.91 (m, ArH), 6.71 (m, ArH), 6.59 (m, ArH), 6.34 (m, ArH), 5.40 (s, OCH_3_) ppm. ^13^C NMR (100 MHz, DMSO-d6) δ = 181.28, 169.78, 164.44, 159.43, 146.12, 145.64, 143.08, 141.06, 140.46, 138.46, 129.34, 124.54, 121.18, 116.08, 114.17, 63.08, 56.24 ppm.

##### Methyl-5-bromo-4’-hydroxy-2,5’-dioxo-1’-phenyl-1’,5’-dihydrospiro[indoline-3,2’-pyrrole]-3’-carboxylate (**4d**)

Yellow solid, 84%,m.p. 244 °C; IR-KBr: 3379.28 (OH), 2950 (CH_3_), 2380 (H-O), 1700 and 1613.20 (C=O), 1383.57 (C-H), 1056.53 (C-N), 550 cm^−1^.^1^H NMR (400 MHz, DMSO-d6) δ = 11.152 (s, 1H, OH), 10.3 (s, 1H, NH) 7.75 (s, 1H, ArH), 7.53 (d, J = 7.4 Hz, 1H, ArH), 7.41 (d, J = 6.3 Hz, 3H, ArH), 7.02 (t, J = 7.1 Hz, 2H, ArH), 6.46 (t, J = 7.3 Hz, 1H, ArH), 3.15 (s, 3H, OCH_3_) ppm. ^13^C NMR (100 MHz, DMSO-d6) δ = 182.14, 169.45, 167.14, 159.81, 154.06, 149.04, 147.04, 145.11, 142.48, 141.71, 140.34, 131.10, 126.79, 124.74, 120.73, 109.44, 75.36, 59.78 ppm.

##### Methyl-5-bromo-1’-(3-chloro-4-fluorophenyl)-4’-hydroxy-2,5’-dioxo-1’,5’-dihydrospiro[indoline-3,2’-pyrrole]-3’-carboxylate (**4e**)

Yellow solid, 74%, m.p. 238–239 °C; IR-KBr: 3150.49 (OH), 2900 (CH_3_), 1748 and 1604.63 (C=O), 1444.26 (C-H), 1384.88 (C-H), 1274 (C-N), 1206.26 (C-O), 1118.65 (C-N), 842.27, 746.68, 678.65, 464.45 cm^−1^. _1_H NMR (400 MHz, DMSO-d6) δ = 11.19 (s, OH), 8.20–8.18 (d, J = 5 Hz, ArH), 7.49–7.44 (t, J = 4.5 Hz, ArH), 7.28–7.24 (m, ArH), 7.12–7.44 (t, J = 4 Hz, ArH), 7.76–7.56 (m, ArH), 6.95–6.93 (d, ArH), 3.86 (s, CH_3_) ppm. ^13^C NMR (100 MHz, DMSO-d6) δ = 186.04, 176.64, 165.07, 159.81, 154.32, 150.43, 147.11, 145.31, 143.56, 142.36, 141.74, 131.13, 127.44, 126.44, 124.75, 109.44, 104.31, 64.11, 59.44 ppm.

##### Methyl-5-bromo-1’-(4-chloro-3-(trifluoromethyl)phenyl)-4’-hydroxy-2,5’-dioxo-1’,5’-dihydrospiro[indoline-3,2’-pyrrole]-3’-carboxylate (**4f**)

Yellow solid, 81%, m.p. 235–236 °C; IR-KBr: 3388.14 (OH), 2950 (CH_3_), 1747.64 and 1610.98 (C=O), 1450 (C-H), 1383.91 (C-H), 1200 (C-O), 1055.65 (C-N), 550 cm^−1^. ^1^H NMR (300 MHz, DMSO-d6) δ = 13.90 (s, 1H), 10.76 (s, 1H), 7.37 (s, 2H), 7.13 (d, J = 8.4 Hz, 1H), 7.01 (s, 1H), 6.98 (d, J = 8.7 Hz, 1H), 3.45(s, 3H) ppm. ^13^C NMR (100 MHz, DMSO-d6) δ = 182.74, 174.16, 163.47, 158.44, 151.47, 148.46, 144.74, 142.91, 140.46, 139.47, 138.47, 128.64, 124.36, 122.41, 121.00, 116.17, 111.46, 58.18, 54.03 ppm.

##### Methyl-4’-hydroxy-5-nitro-2,5’-dioxo-1’-phenyl-1’,5’-dihydrospiro[indoline-3,2’-pyrrole]-3’-carboxylate (**4g**)

Yellow solid, 84%, m.p. 246 °C; IR-KBr: 3314.75 (OH), 2930 (CH_3_), 2300 (H-O), 1740.47 and 1615.94 (C=O), 1526.9 (C-H), 1384.15 (C-H), 1202.56 (C-O), 1072.05 (C-N), 751.05 cm^−1^. ^1^H NMR (800 MHz, DMSO-d6) δ = 11.19 (s, 1H, OH), 8.20–8.19 (d, J = 8.1 Hz, ArH), 7.95–7.93 (d, J = 16 Hz, ArH), 7.89–7.88 (d, J = 8 Hz, ArH), 7.75–7.68 (t, J = 8 Hz, ArH), 7.67–7.56 (m, ArH), 7.60 (s, ArH) 7.46–7.44 (t, J = 16 Hz, ArH), 7.10–7.08 (t, J = 8 Hz, ArH), 6.95–6.94 (d, J = 8 Hz, ArH), 3.34 (s, OCH3) ppm. ^13^C NMR (100 MHz, DMSO-d6) δ = 179.83, 166.49, 161.67, 154.66, 149.36, 144.12, 142.33, 139.64, 138.46, 136.52, 134.11, 130.34, 121.82, 120.47, 119.34, 118.12, 111.46, 78.46, 58.18 ppm.

##### Methyl-1’-(3-chloro-4-fluorophenyl)-4’-hydroxy-5-nitro-2,5’-dioxo-1’,5’-dihydrospiro[indoline-3,2’-pyrrole]-3’-carboxylate (**4h**)

Light yellow solid, 71%, m.p. 241 °C; IR-KBr: 3341.44 (OH), 2950 (CH3), 1726.07 and 1614.12 (C=O), 1454.87 (C-H), 1338.28 (C-H), 1160.55 (C-O), 1074.13 (C-N), 765.23 cm^−1^. ^1^H NMR (800 MHz, DMSO-d6) δ = 11.70 (s, OH), 11.67 (s, 1H, NH), 8.56 (d, J = 7.9 Hz, ArH), 8.13–8.30 (t, J = 7.8 Hz, ArH), 8.30 (s, ArH), 7.16–6.64 (m, ArH), 3.25(s, OCH3) ppm. ^13^C NMR (800 MHz, DMSO-d6) δ = 172.95, 167.70, 161.72, 150.77, 150.07, 143.36, 128.84, 128.56, 125.95, 125.73, 112.24, 119.75, 117.43, 112.53, 111.34, 81.29, 21.02 ppm. HRMS (ESI-TOF *m/z*) (M + H) + calculated for C_19_H_12_ClFN_3_O_7_ + 449.00784, found 448.0347; 449.0382; 450.0327; 451.0342; 452.0451, (M + Na) + calculated for C_19_H_11_ClFN_3_NaO_7_ + 470.28976, found 470.0175; 471.0194; 472.014.

##### HPLC Analysis of SOX **4a**

The purity of the synthesized compound, used for in vitro cell culture studies, was assessed through HPLC at Integral University, Lucknow. Briefly, 20 µL SOX **4a** was analyzed using Shimadzu 20AD Gradient LC System with PDA Detector system. A C-18 column (4.6 mm × 250 mm, 5 μm) was used to achieve the chromatographic separation. The mobile phase comprising methanol: H_2_O (70:30, *v/v*) was applied at a flow rate of 0.5 mL/min. The SOX **4a** was detected at 254 nm with a retention time of 7.928 min in the HPLC system thermo-stated at 25 °C.

### 2.2. Computational Chemistry

#### 2.2.1. Substituted Spirooxindole-Pyrrolines (**4a**–**h**) Exert Drug-like Properties

The drug discovery program (DDP) involves the assessment of a set of distinct factors to identify the drug-likeness of a chemical entity. To date, various artificial intelligence (AI)-based strategies, i.e., absorption, distribution, metabolism, excretion, and toxicology (ADMET) and drug-likeness are currently being exploited as a part of the DDP to avoid unnecessary wasting of time, budget, and manpower [46,47,48]. In the same context, we also implied AI-based approaches, i.e., analysis of Lipinski’s rule of five and ADME to assess the drug-like properties of our newly synthesized spirooxindole-pyrrolines (**4a**–**h**). Lipinski’s rule of five includes some physicochemical descriptors that each and every chemical entity should follow in order to be qualified as an appropriate therapeutic agent. These descriptors include the molecular weight (M.W. < 500 Da), H-bond donors (HBDs: <5), H-bond acceptors (HBAs: <10), rotatable bonds (<10), topological polar surface area (TPSA) not exceeding the thresholds of 140 Å^2^ and octanol-water partition coefficient (Log P) not exceeding the value of 5 [48,49]. The initial drug-likeness analysis depicted that all the newly synthesized spirooxindole-pyrrolines (**4a**–**h**) fall under the acceptable scores of Lipinski’s rules of five if only one violation was permitted (Supplementary Table S1). Briefly, the M.W. for all the substituted spirooxindole-pyrrolines was found to be less than 500 except **4f**, which had a M.W. of 531.66, whereas the number of HBDs and HBAs for **4a**–**h** were recorded in the desirable thresholds of less than 5 and 10, respectively. On the other hand, the number of rotatable bonds was also found to be either three or four for all the substituted spirooxindole-pyrrolines (**4a**–**h**), which falls within the permissible thresholds of less than five [48].

On the other hand, considering the potential of hydrophobicity in drug distribution patterns, analysis of Log P has widely been used to assess the permeability of drug candidates, whereas the TPSA is used as the measure of the polarity and trans-membrane transport of compounds [50,51]. The results of our Log P and TPSA analysis showed that all the synthesized spirooxindole-pyrrolines (**4a**–**h**) have desirable Log *p* values (Log *p* ≤ 5, ranging from 1.48 for **4g** to 2.91 for **4f**). Moreover, the values for TPSA were also within the permissible thresholds (<140 Å^2^) except for the two spirooxindole-pyrrolines, i.e., **4g** and **4h,** where each of the here-mentioned spirooxindole-pyrroline exhibited TPSA > 140 Å^2^ (141.76 Å^2^ for each); however, these violations (slightly higher TPSA) were not significant enough to rule out these newly synthesized spirooxindole-pyrrolines from this study. These values for the TPSA for all the newly synthesized spirooxindole-pyrrolines (**4a**–**h**) were similar to that reported in previously published reports [48,52]. These properties signify the fact that the newly synthesized spirooxindole-pyrrolines qualify for all the tests for drug-likeness and can further be assessed for other pharmacological effects.

#### 2.2.2. Substituted Spirooxindole-Pyrrolines (**4a**–**h**) Have Acceptable ADME Properties

The prediction of distinct pharmacological measures, i.e., ADME through AI-based in silico strategies, is the most fundamental and persuasive step in the screening of chemical libraries for further drug discovery [46,48]. Our ADME analysis depicted that the newly synthesized spirooxindole-pyrrolines exert varying aq. solubility ranging from 0.330 to 100.057 mg/L for **4f** and **4c**, respectively (Supplementary Table S2). However, the reference anticancer agent, doxorubicin, had the greatest aq. solubility (112.691 mg/L). On the other hand, blood-brain barrier (BBB) penetration scores for all the substituted spirooxindole-pyrrolines (**4a**–**h**) were less than 2 (0.013 to 0.623 C. Brain/C. Blood). Most importantly, the substituted **4h** showed the lowest affinity against the BBB (BBB score: 0.013 C. Brain/C. Blood) as chemical entities with a BBB score > 2.0, between 2.0~0.1, and <0.1 correspond to the highest, moderate, and the lowest absorption across the BBB, respectively [48,53,54]. To sum up, all the substituted spirooxindole-pyrrolines (**4a**–**h**) exhibited either moderate or low transport against the BBB.

On the other hand, the assessment of Caco-2 and MDCK cell permeability has been established as the crucial measure of the DDP [54,55,56]. The Caco-2 and MDCK cell permeability was predicted through PreADMET. Our results from PreADME analysis showed that the substituted spirooxindole-pyrrolines (**4a**–**h**) exhibited a Caco-2 permeability range of 19.275 for **4h** to 21.090 for **4g** nm/s, which was suggestive of the fact that all of these newly synthesized spirooxindole-pyrrolines (**4a**–**h**) exert moderate permeability against Caco-2 cells. Similarly, the MDCK cell permeability score ranged from 0.1555 for **4f** to 9.255 for **4c** nm/s, which signifies that all the substituted spirooxindole-pyrrolines synthesized in the current study exhibited lower permeability across MDCK cells (score < 4.0) except **4c** which showed a moderate permeability across these cells (score 9.255 nm/s). The permeability scores ≤ 4, >4–70, and >70 (nm/s) are suggestive of low, middle, and high permeability, respectively, in various cellular models [48,57].

Besides the permeability across the BBB, Caco-2, and MDCK cells, evaluation of the permeability across the skin/epidermal linings is thought to be of great importance when it comes to the transdermal drug delivery as well as the unintended exposure to harmful chemical entities [48,58]. In this regard, the skin permeability score (Log Kp) for the newly synthesized spirooxindole-pyrrolines was reported to be negative (from −2.723 to −4.233 cm/h for **4f** and **4b**, respectively), which is considered the acceptable range of Log Kp for therapeutic agents [59]. The fate of the drugs beyond absorption into the blood to the target tissues is governed by a set of factors (i.e., high M.W., solubility, and HBAs/HBDs) but plasma protein binding (PPB), perhaps, has been established amongst the major detrimental factors [48]. Chemical species with a very high PPB score tend to have comparatively higher persistence in the circulation or an elongated plasma half-life (t_1/2_). More importantly, high PPB efficiency also impacts drug efficacy as usually the circulating fraction of drugs is accredited to the potent pharmacological actions [60,61]. In the same vein, the PPB for substituted spirooxindole-pyrrolines (**4a**–**h**) ranged from 89.411 to 92.711% for **4b** and **4h**, respectively. In contrast, doxorubicin exerted comparatively lower PPB efficiency (only 32.789%), which could be associated with poor absorption/distribution as well as its limited efficiency in the management of different malignancies [62].

Human intestinal absorption (HIA), a key determinant of drug suitability in the current drug discovery program, is derived from the bioavailability, absorption, and excretion of the drugs [48,49,63]. The establishment of different cancers and associated complications are attributed to distinct factors, i.e., reduced apoptosis, the state of oxidative imbalance, inflammatory cascades, and the accumulation of distinct advanced glycation end products (AGEs) in the circulation as well as various tissues/organs [31,64,65,66]. Therefore, the therapies advised against distinct malignancies should not only be focused on the regulation of the apoptosis or cell cycle arrest but also on ameliorating the here-mentioned multiple targets residing either in the circulation or various tissues/organs [67,68]. In this regard, a pleiotropic drug with ameliorative efficacy against all these ghosts must have enhanced absorption as well as bioavailability in the circulation and other target tissues.

In the same context, the HIA for all the substituted spirooxindole-pyrrolines (**4a**–**h**) in our study ranged from 81.85% for **4g** to 95.58% for **4f**. Most strikingly, HIA for all the spirooxindole-pyrrolines ranged from 90–95%, except **4g**, which showed an HIA score of 81.85% only. The high HIA scores of substituted spirooxindole-pyrrolines synthesized in the current study are advocating the fact that these substituted derivatives (**4a**–**h**) may have potent pharmacological efficacies owing to their elevated HIA as well as bioavailability. In contrast, the standard antiproliferative drug, doxorubicin, recorded a very poor HIA score of 31.95%, and such a poor bioavailability and absorption alone has been linked with the compromised anticancer efficacy of this commercially available drug [62]. Even the previous pharmacokinetic evaluations reported that the maximum plasma concentration (C_max_) for doxorubicin was found to be only 29.2, 75.0, and 142.0 ng/mL when orally administered in rats at the dosage of 20, 50, and 100 mg/kg, respectively [62].

Moreover, the cytochrome P450 2D6 (CYT.p450 2D6 or CYP2D6) has been associated with around 25% of the drug metabolism in various tissues, organs, and circulation, as well as excretion from the body [48,69]. The fate of CYP2D6-directed drug metabolism differs from person to person as they may behave either as ultra-metabolizers or poor metabolizers of drugs, where the former suffer from the poor availability of drugs while the latter are supposed to be challenged by the compromised clearance from the body [69,70], therefore, making CYP2D6-directed drug metabolism a detrimental factor in dose selection criteria [48,71]. The results from this study showed that all the substituted spirooxindole-pyrrolines (**4a**–**h**) neither behaved as CYP2D6 inhibitors nor CYP2D6 substrates, which signified the fact that the metabolism of these substituted spirooxindole-pyrrolines (**4a**–**h**) undergoes more or less in a CYP2D6-independent manner. 

In this case, their potent pharmacological effects may be attributed to their higher PPB ability (>90%), which makes them more persistent as well as more bioavailable and also delays their instant removal from the circulation. In addition, none of the newly synthesized spirooxindole-pyrrolines (**4a**–**h**) acted as CYP2D6 substrates, which also reflects their persistent nature as well as enhanced therapeutic efficacy once these are administered in the body. These corroborations are well supported by recent reports [48,69,72].

### 2.3. Newly Synthesized SOXs Exhibit Strong Anticancer Activity via Targeting Multiple Enzymes: Outcomes from the In Silico Molecular Docking Studies

#### 2.3.1. Substituted SOXs (**4a**–**h**) Are Potent Inhibitors of CD44

Previous studies have confirmed the association of CD44 with various malignancies, including prostate cancer [3,4]. In an attempt to screen the substituted SOXs for their ability to occupy the binding pocket of CD44, the PyRx, a web-based server for virtual screening of drug-like candidates, was implied. As a result, all the SOXs exhibited significant interaction against the active pocket of CD44, and their binding score ranged from −6.5 to −7.4, whereas SOX **4a** showed the best binding affinity with the least negative binding score (−7.4) (Table 3). 

**Figure 2:**
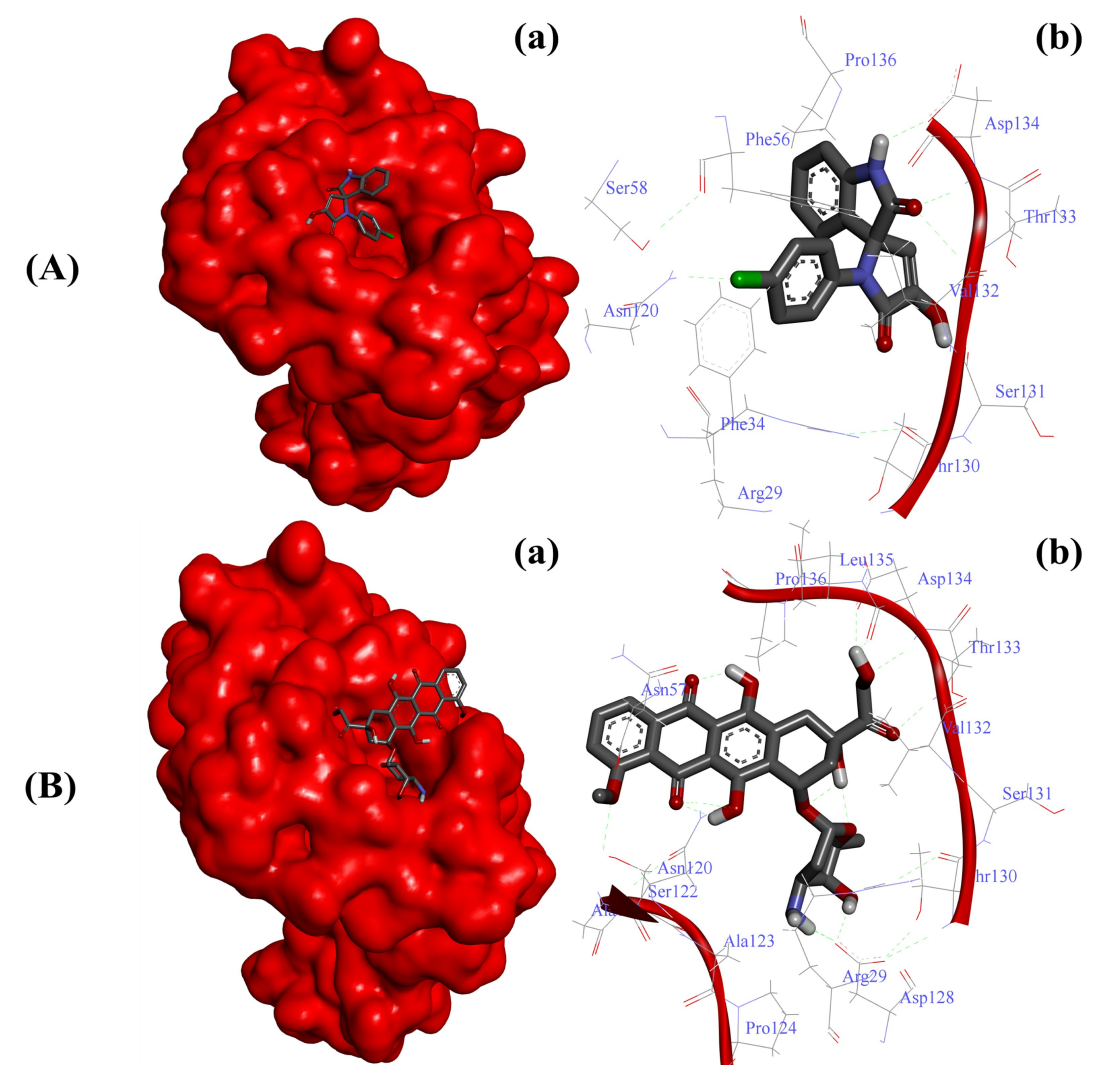
Substituted SOX (**4a**) is a potent inhibitor of human CD44 (PDB ID: 1UUH). (**A**) Binding pattern of the substituted SOX (**4a**) against human CD44 (∆G: −6.65 Kcal/mol); (**B**) Binding pattern of doxorubicin against human CD44 (∆G: −5.83 Kcal/mol). Subpanels (**a**) represent the interaction of 4a and doxorubicin inside the active pocket of the target protein (represented as a red surface). Subpanels (**b**) show the 2D image of the interacting residues of the CD44 with our drugs.

On the other hand, the interaction of doxorubicin with CD44 was not as strong as reported in the case of substituted SOX **4a**, as evident by its slightly higher binding score (binding score: −7.0). Moreover, the strong interaction of the best SOX (**4a**) against CD44 was further validated through Autodock 4.0, which revealed that 4a strongly interacted with the active pocket of CD44 with a binding energy (∆G) of -6.65 Kcal/mol (Table 4). The binding of **4a** was facilitated by the involvement of Arg29, Phe34, Phe56, Asn120, Thr130, Ser131, Val132, Thr133, Asp134, Pro136, and Ser158 residues of CD44 (Figure 2A). In contrast, this interaction of **4a** with CD44 was better than that reported in the case of doxorubicin (∆G: −5.83Kcal/mol). Moreover, the doxorubicin-CD44 complex was stabilized by the interaction of Arg29, Asn57, Asn120, Ala121, Ser122, Ala123, Pro124, Asn128, Thr130, Ser131, Val132, Thr133, Asp134, Leu135, and Pro136 residues residing in the active pocket of CD44 (Figure 2B).

#### 2.3.2. Substituted SOXs (**4a**–**h**) Are Potent Inhibitors of the Tyrosine Kinase Domain of the EGFR

**Figure 3:**
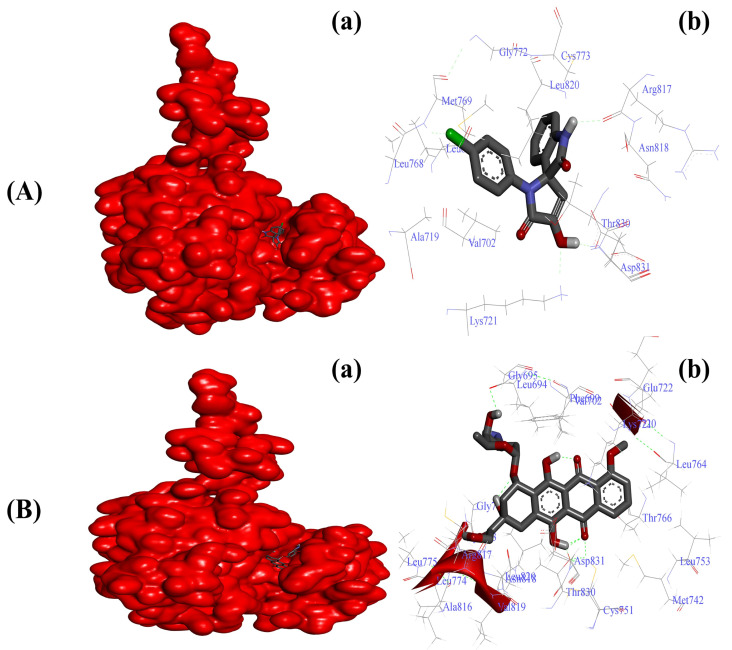
Substituted SOX (**4a**) is a potent inhibitor of the tyrosine kinase domain of the EGFR (PDB ID:1M17). (**A**) Binding pattern of the substituted SOX (**4a**) against human EGFR (∆G: −6.55 Kcal/mol); (**B**) Binding pattern of doxorubicin against human CD44 (∆G: −10.11 Kcal/mol). Subpanels (**a**) represent the interaction of 4a and doxorubicin inside the active pocket of the target protein (represented as a red surface). Subpanels (**b**) show the 2D image of the interacting residues of the EGFR with our drugs.

Various researchers have reported overexpression as well as enhanced functionality of EGFR in tumor tissues [21,22]. Our initial virtual screening through PyRx identified SOX **4a** as the best inhibitor of the tyrosine kinase domain of EGFR, with a binding score of −8.7 among other newly synthesized SOXs (**4a**–**h**). The reference standard doxorubicin also formed a stable doxorubicin–EGFR complex with a better binding score of −10.1 Kcal/mol (Table 3). The inhibitory effect of the substituted SOX with the best binding score against EGFR (**4a**) was further validated through a deep interacting analysis using Autodock 4.0. The result from our docking studies showed that the substituted **4a** formed a stable complex with EGFR with a ∆G score of −6.55 Kcal/mol (Table 4), which falls within the ideal interaction energy as ∆G anything less than −4.0 Kcal/mol is considered a substantial interaction [73,74]. This 4a–EGFR complex was stabilized by the interaction of Leu694, Val702, Ala720, Lys721, Leu768, Met769, Gly772, Cys773, Arg817, Asn818, Leu820, Thr830, and Asp831 residues of EGFR (Figure 3A). In contrast, the doxorubicin–EGFR complex showed a ∆G of −10.11 Kcal/mol, and the formation of this complex became feasible due to the involvement of Leu694, Phe699, Ala719, Lys721, Glu738, Met742, Val762, Leu764, Thr766, Gln767, Leu768, Met769, Pro770, Gly772, Arg817, Asn818, Leu820, Thr830, Asp831, and Phe832 residues of the EGFR (Figure 3B).

#### 2.3.3. Substituted SOXs (**4a**–**h**) Inhibit the Activity of 5-β-Reductase (AKR1D1)

**Figure 4:**
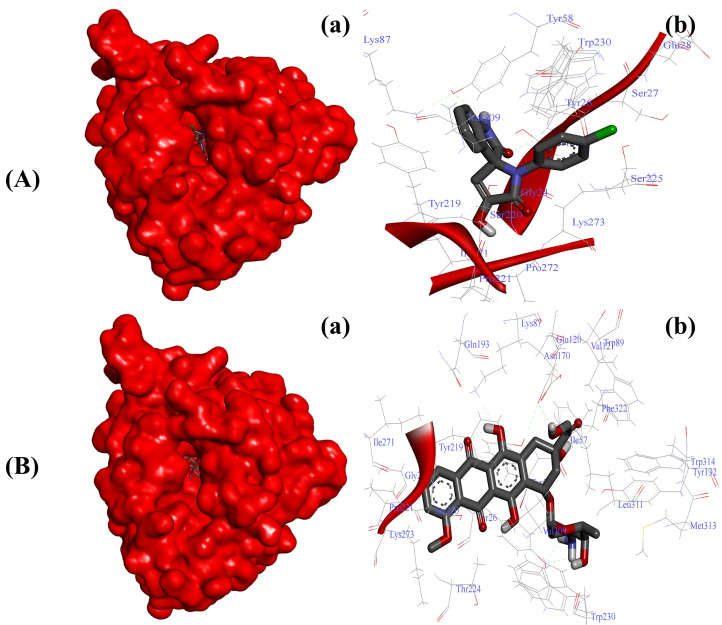
Substituted SOX (**4a**) inhibits the activity of 5-β-reductase/AKR1D1 (PDB ID: 3CAQ). (**A**) Binding pattern of the substituted SOX (**4a**) against human AKR1D1 (∆G: −8.73 Kcal/mol); (**B**) Binding pattern of doxorubicin against human CD44 (∆G: −10.01 Kcal/mol). Subpanels (**a**) represent the interaction of **4a** and doxorubicin inside the active pocket of the target protein (represented as a red surface). Subpanels (**b**) show the 2D image of the interacting residues of the AKR1D1 with our drugs.

Considering the impact of AKR1D1 in cancer metabolism [14,16], we initially screened the ability of our newly synthesized SOXs (**4a**–**h**) for their potential ability to occupy the active pocket of this enzyme through the PyRx server. At this attempt, all the substituted SOXs (**4a**–**h**) showed substantial interaction within the binding pocket of AKR1D1, while their binding scores ranged between −7.1 to −10.0 (Table 3). Among all the substituted SOXs, **4a** showed the best interaction with AKR1D1 with a binding energy score of −10.1, whereas doxorubicin also showed significant interaction with AKR1D1 (binding score: −10.4). The best interacting SOX (**4a**) was further subjected to the deep interaction analysis via using Autodock 4.0 (Table 4), which revealed that it strongly interacts with the Gly24, Thr25, Tyr26, Ser27, Glu28, Tyr58, Lys87, Tyr219, Ser220, Pro221, Ser225, Tyr230, Ile271, Pro272, Gly273, and Val309 residues of the active pocket of AKR1D1 (∆G: −8.73 Kcal/mol) (Figure 4A). 

On the other hand, doxorubicin also showed a greater binding affinity for AKR1D1 with a ∆G of −10.01 Kcal/mol and the involvement of Gly24, Tyr26, Ile57, Tyr58, Lys87, Trp89, Val121, Glu120, Tyr132, Asn170, Gln193, Tyr219, Ser220, Pro221, Thr224, Trp230, Ile271, Lys273, Val309, Leu311, Met313, Trp314, and Phe322 residues (Figure 4B).

#### 2.3.4. Substituted SOXs (**4a**–**h**) Occupy the Binding Pocket of HER-2

**Figure 5:**
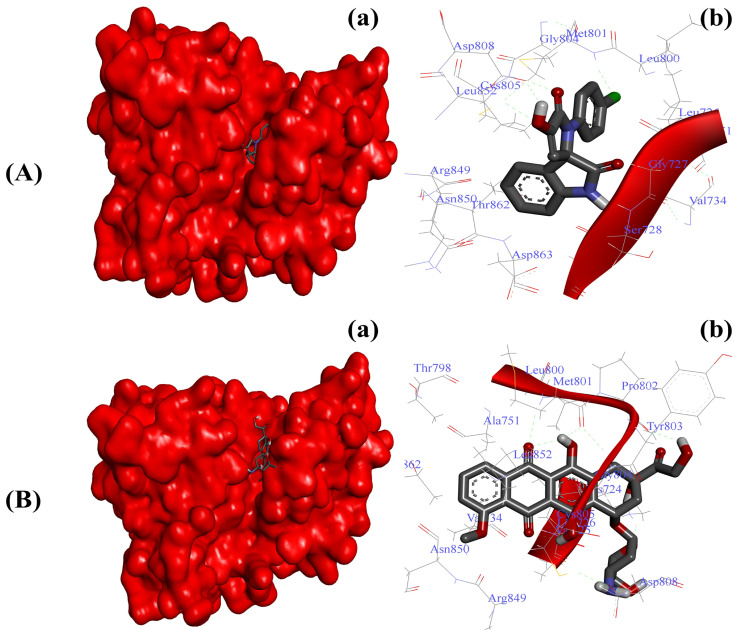
Substituted SOX (**4a**) occupies the binding pocket of HER-2 (PDB ID: 3PP0). (**A**) Binding pattern of the substituted SOX (**4a**) against human AKR1D1 (∆G: −7.27 Kcal/mol); (**B**) Binding pattern of doxorubicin against human CD44 (∆G: −8.94 Kcal/mol). Subpanels (**a**) represent the interaction of **4a** and doxorubicin inside the active pocket of the target protein (represented as a red surface). Subpanels (**b**) show the 2D image of the interacting residues of the HER-2 with our drugs.

Blocking the enhanced activity of HER-2 has been proven as a promising anticancer strategy in the last few decades [7,8]. To further validate the antiproliferative effects, we screened our newly synthesized SOXs (**4a**–**h**) for their ability to inhibit the active pocket of HER-2 via implying the PyRx server. In this attempt, the SOXs (**4a**–**h**) occupied the binding pocket of HER-2 (binding score: −6.6 to −7.8) with the greatest affinity exhibited by **4a** (binding score: −7.8), whereas doxorubicin also exhibited a comparable binding score (−7.7) (Table 3). Subsequent detailed molecular docking analysis through Autodock 4.0 also confirmed the strong binding of substituted SOX (**4a**) against HER-2 (Table 4). The **4a**–HER-2-complex was stabilized by the involvement of Gly77, Ser78, Lue726, Gyl729, Val734, Ala751, Leu800, Met801, Gly804, Cys805, Asp808, Asp850, Leu852, Arg859, Thr862, and Asp863 amino acid residues and potent binding energy (∆G: −7.27 Kcal/mol) (Figure 5A). On the other hand, doxorubicin exerted stronger binding with HER-2 (∆G: −8.94 Kcal/mol) when compared to the ∆G of substituted SOX (**4a**). The interaction of doxorubicin with HER-2 was supported by different amino acid residues of HER-2 including Lys724, Val725, Leu726, Val734, Ala751, Thr798, Leu800, Met801, Pro802, Tyr803, Gly804, Cys805, Asp808, Arg849, Asn850, Leu852, and Thr862 (Figure 5B).

### 2.4. Substituted SOX (**4a**) Exerts Antiproliferative Effects against Prostate Adenocarcinoma Cells (PC-3)

#### 2.4.1. The Substituted SOX (**4a**) Exhibits Cytotoxic Effects on PC-3 Cells

**Figure 6:**
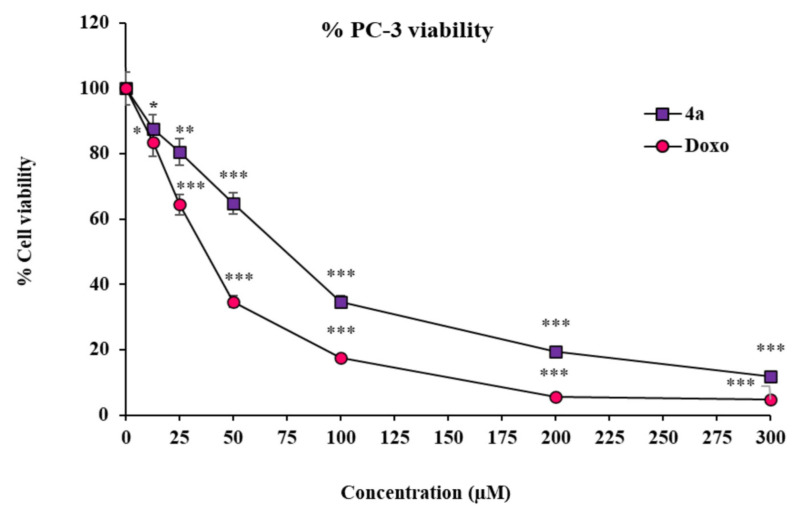
Cytotoxicity of **4a** and doxorubicin against PC-3 cells. Values (percent cell viability) are mean ± SEM from triplicate measurements. Significantly different from untreated PC-3 cells at *** *p* < 0.001; ** *p* < 0.01; and * *p* < 0.05. The statistical analysis (ANOVA) was performed using GraphPad Prism Software version 9.5.0., San Diego, CA, USA.

To corroborate our findings from the in silico molecular modeling studies, we further opted for distinct strategies implying PC-3 cells as a prostate cancer model. The very first among those strategies was the assessment of the cytotoxicity effect of the above-screened best in silico modulator (**4a**) of multiple enzymes/biomarkers specific to the cancer establishment, i.e., CD44, EGFR, AKR1D1, and HER-2. The in vitro cytotoxicity effect of substituted SOX **4a** was determined using an MTT assay, the most widely used assay to observe the toxicity of the test drugs in cell culture settings [66]. In this attempt, the substituted SOX (**4a**) was found to have substantial cytotoxicity against PC-3 cells in a dose-dependent fashion (IC_50_: 72.51 ± 2.35 µM) (Figure 6). Moreover, the cytotoxicity effect of substituted **4a** was further compared to the effect that was reported in the case of the reference standard doxorubicin, which showed an IC_50_ of 37.90 ± 2.10 µM. These findings are well supported by the previous report, which also showed similar cytotoxicity effects of doxorubicin on PC-3 cells [75].

**Figure 7:**
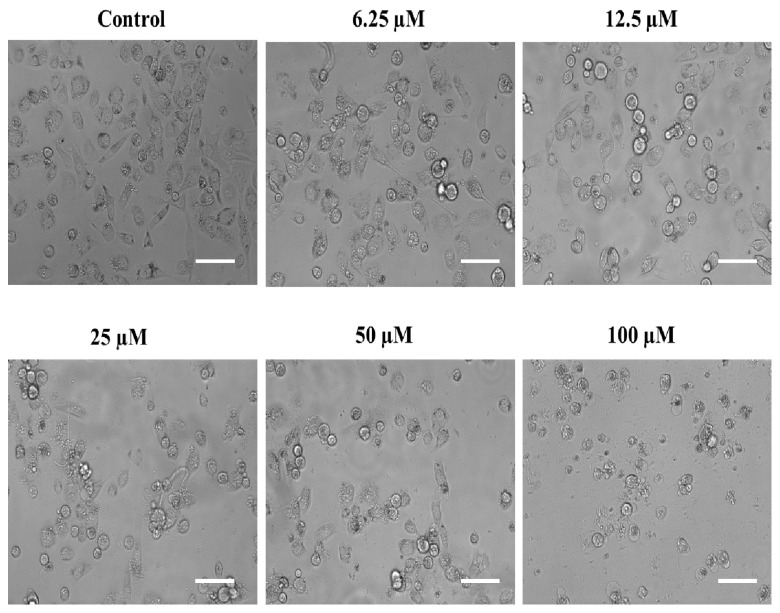
Phase contrast micrographs showing the impact of substituted SOX (**4a**) on the morphological features of PC-3 cells after 24 h. For the assessment of morphological features, an equal population of the PC-3 cells was seeded in the 96-well plate and incubated with varying doses of **4a** (0, 6.25, 12.5, 25, 50, and 100 μM). Following the incubation period, photomicrographs were taken using a FLoid^TM^ Imaging Station, Thermo Fisher Scientific, Waltham, MA, USA. Scale bar: 100 μM.

Usually, all cells have their own characteristic cytological features and morphologies, and so do the cancerous cells. In most cases, the cancerous cells are distinguishable from the noncancerous ones due to their comparatively larger nucleus as well as irregular morphologies [76]. However, these characteristic features may be altered when these cells are exposed to chemotherapeutic agents. In the same context, we also assessed the possible aberrations in the cytomorphological characteristics of the androgen-independent PC-3 cells after they were exposed to the above-screened substituted SOX (**4a**). In this attempt, our phase contrast microscopic analysis revealed that exposure to different dosages of substituted SOX (**4a**) resulted in diverse morphological aberrations in the PC-3 cells that were easily characterized in terms of cellular size, cytoplasmic abnormalities, nuclear condensation, granule formation as well as dispersed cellular counterparts (Figure 7). These morphological abnormalities, together with the cytotoxic effects on PC-3 cells (evident through MTT assay), are indicative of the substantial antiproliferative potential of substituted SOX (**4a**).

#### 2.4.2. The Substituted SOX (**4a**) Triggers ROS Generation in PC-3 Cells

**Figure 8:**
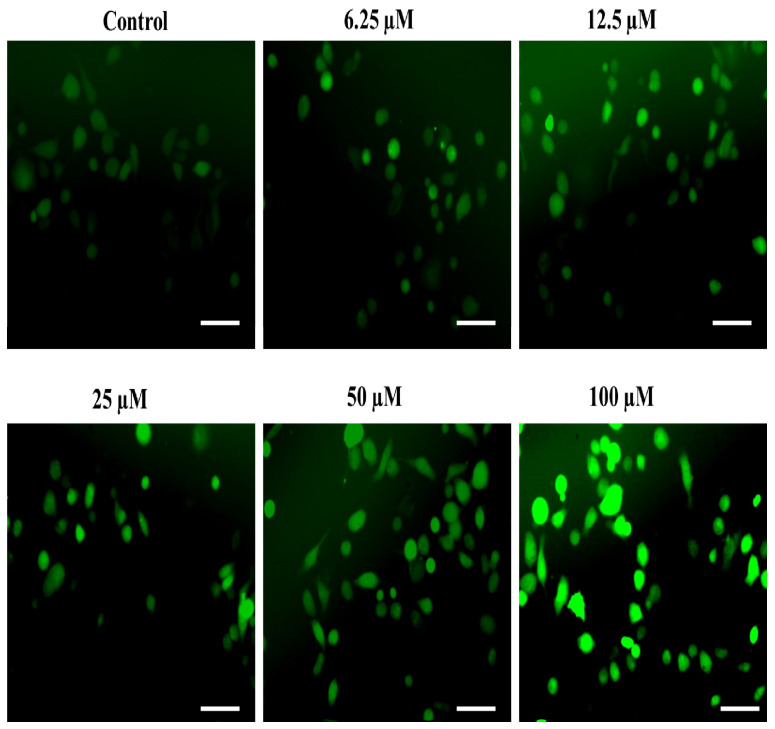
The substituted SOX (**4a**) triggers ROS generation in PC-3 cells**.** For the detection of ROS-specific fluorescence, PC-3 cells seeded in each well were treated with 0.0 to 100 μM substituted SOX (**4a**) for 24 h, and the cells were then stained with 10 µM DCFH-DA and incubated for half an hour and imaging was done using a FLoid^TM^ imaging station, Thermo-Scientific, Waltham, MA, USA. Scale bar: 100 μM.

An imbalanced redox state is well-reckoned to cause the establishment of distinct non-communicable ailments such as atherosclerosis [26,73,77], neuro-degeneration [29], hyperglycemia [30,78] and associated complications, particularly nephropathy [32]. Interestingly, the ability of robust ROS generation is thought to be the key mechanism behind the antiproliferative efficacy of the majority of the commercially available chemotherapeutic agents [79]. In the same context, we also assessed the impact of our substituted SOX, i.e., **4a**, on ROS generation as a probable mechanism behind its ability to negatively affect the survival of PC-3 cells. The observations from the fluorescence microscopic analysis demonstrated that incubation with substituted **4a** led to robust ROS generation in PC-3 cells (Figure 8). This phenomenon was amplified with an increase in the dosages of **4a** (6.25 to 100 µM). Such robust ROS production by the exposure of **4a** may be attributed to its potent chemotherapeutic effect on PC-3 cells as ROS are well known to trigger distinct injuries to the cells, including damage to the cellular macromolecules, membrane oxidation and inflammatory signaling [27,28,66,80,81]. These exciting findings are strongly advocating the chemotherapeutic potential of substituted SOX (**4a**).

#### 2.4.3. The Substituted SOX (**4a**) Stimulate the Nuclear Condensation in PC-3 Cells

**Figure 9:**
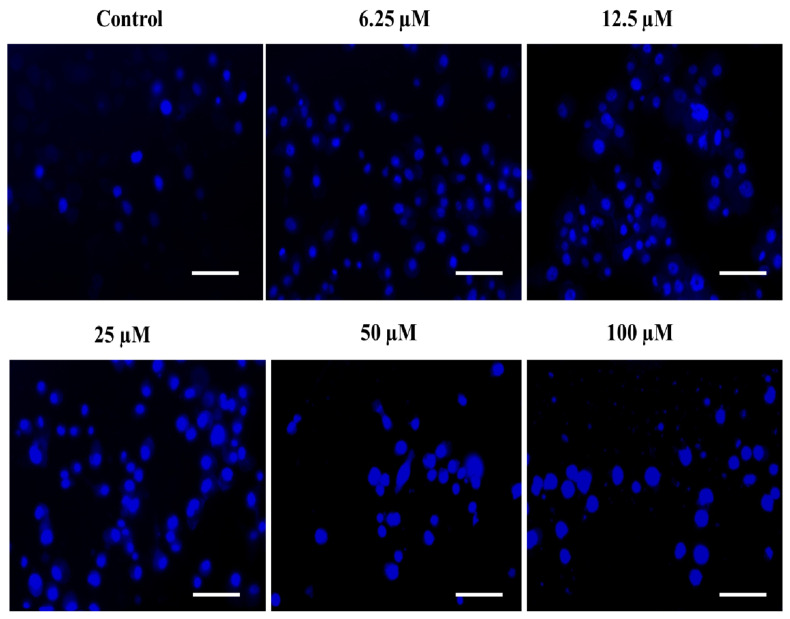
The substituted SOX (**4a**) stimulates nuclear condensation/apoptosis in PC-3 cells. The PC-3 cells were challenged with varying doses of **4a** (0.0–100 μM) and incubated for 24 h and then stained with DAPI, and the DAPI-specific fluorescence was captured using a blue filter in a FLoid^TM^ Imaging station (Thermo-Scientific, Waltham, MA, USA). Scale bar: 100 μM.

The rationale behind the success of most of the commercially implied chemotherapeutic drugs in the last few decades is thought to be their capability to induce apoptosis in cancerous cells. The phenomenon of apoptosis is characterized by a set of key events, including the shrinkage of the nucleus, condensation of chromatin, DNA cleavage/fragmentation, the appearance of membrane protrusions (or cytoplasmic blebs), and the formation of apoptotic bodies [82,83]. Therefore, the assessment of nuclear fragmentation/condensation has been established as one of the most preferred strategies to screen the efficacy of the test chemotherapeutic agents. In this order, we also determined the effect of the substituted SOX (**4a**) on the apoptosis-like phenomenon in PC-3 cells and observed that the treatment with **4a** significantly triggered the nuclear condensation/fragmentation as evident by the enhanced DAPI-specific fluorescence in a dose-dependent fashion against an untreated control (Figure 9). This enhancement in the DAPI-specific fluorescence after **4a** treatment could be attributed to the ability of this fluorophore to combine within the A=T rich milieu of the minor groove of the fragmented/condensed DNA, and the same has already been established by previous reports [83,84]. These findings are clearly indicating the chemotherapeutic potential of the substituted **4a** via induction of ROS generation as well as nuclear condensation, which ultimately led to cell death or apoptosis in PC-3 cells.

## 3. Materials and Methods

### 3.1. Chemistry

#### 3.1.1. Synthesis of the SOX Derivatives (**4a**–**h**)

The reaction was initiated by mixing isatin (1 mM), 4-chloro-3-(trifluoromethyl)aniline (1 mM), and dimethyl acetylenedicarboxylate (1 mM) in 5 mL of different solvents (i.e., ethanol, MeOH, EtOAc, acetone, DMSO, and isopropanol) in the presence of *p*-TSA (0.0, 0.25, 0.50, and 0.75 mM; catalyst) followed by stirring the mixture at room temperature for varying durations (6–16 h) (Scheme 1). Following the designated optimization steps with the above-mentioned three components, the concentrated precipitates were collected and washed with a respective solvent in order to obtain pure compounds devoid of *p*-TSA. On the basis of the findings from these optimizations hits, we concluded that the optimal conditions for the synthesis of the substituted spirooxindole pyrroline (**4a**) were *p*-TSA: 0.5 mM and solvent: EtOH. The rest of the compounds (**4b**–**h**) were synthesized by using distinct isatin derivatives, aryl amine derivatives, and dimethyl acetylenedicarboxylate (remained constant) in the presence of 0.5 mM *p*-TSA and ethanol at RT.

#### 3.1.2. Apparatus Used for the Characterization

The melting point of all the synthesized compounds was determined with the help of the open capillary tube method using IA 9100 MK-Digital uncorrected melting Point analyzer Griffin Apparatus at the Department of Chemistry, Integral University. Infrared Spectroscopic analysis was done on Agilent Cary 630 FT-IR Spectrometer (Range: 4000–450 cm^−1^) Perkin-Elmer Spectrum version 10.03.06 at CSIR-CDRI, Lucknow, and the spectra were expressed as wave number (cm^−1^) with KBr discs. ^1^H NMR Spectra was determined using Advance-400/800 MHz Bruker, Switzerland, and Bruker AVLL-300/800 MHz (Bruker, Fällanden, Switzerland) using DMSO-d_6_ as a solvent. 

The chemical shifts were denoted by δ ppm units using trimethylsilane as the internal standard. ^13^C NMR spectra were recorded using Bruker AVLL-100 MHz (Bruker, Switzerland) using DMSO-d_6_. High-Resolution Mass Spectra were taken on Acquisition SW-6200 series TOF, version Q-TOF B.05.00 at CBMR, SGPGIMS, Lucknow. The completion of all the reactions was monitored with the help of thin-layer chromatography (TLC) and using variable proportions of ethyl acetate and petroleum n-hexane.

### 3.2. Computational Chemistry

#### 3.2.1. Ligand Preparation

The two-dimensional structures of synthesized SOX derivatives were drawn with the help of ChemDraw Professional v15.1 and subjected to prepare a three-dimensional structure with the help of Chem3D v15.1. The structural modification, geometrical correction, and optimization were performed with the help of Merck Molecular Force Field (MMFF94). Protoss is an online tool that automatically predicts hydrogens for the interaction between protein-ligand complexes (https://proteins.plus/), accessed on 5 February 2022. The substituted SOXs were subjected to a single-step minimization by the steepest descent method for 500 steps and an RMS gradient of 0.01 [85]. To perform docking analysis, the eutectic state of protonation of the ligands was found at pH 7.4.

#### 3.2.2. ADME and Drug-Likeness of Substituted SOXs

The pharmacokinetic details of the substituted SOXs were obtained from an online ADME tool, and the drug-likeness of the newly synthesized SOXs was analyzed through the SwissADME tool (http://www.swissadme.ch); accessed on 15 February 2022 [49].

#### 3.2.3. Retrieval of the Human CD-44, EGFR, AKR1D1, and HER-2

The 3D structures of human CD-44, EGFR, AKR1D1, and HER-2 were taken from the PDB (http://www.rcsb.org/) (accessed on 21 February 2022) using the PDB IDs 1UUH, 1M17, 3CAQ, and 3PP0, respectively, and visualized through BIOVIA Discovery Studio Visualizer 2020. These proteins had the resolutions of 2.2 Å, 2.6 Å, 2.2 Å, and 2.25 Å, respectively, and were processed as described earlier to remove the co-crystalized chemical entities [27,74]. The steepest descent and conjugate gradient approaches were implied for the energy minimization of the above-mentioned targets involved in the cancer progression. The human CD-44, EGFR, AKR1D1, and HER-2 were subjected to the DEEPSITE (available at: https://www.playmolecule.com/), accessed on 5 March 2022, for the prediction of the binding pockets [86]. 

#### 3.2.4. Molecular Docking of Substituted SOXs against CD-44, EGFR, AKR1D1, and HER-2

The newly synthesized SOXs (**4a**–**h**) were firstly subjected to PyRx to screen the best inhibitor of the hyaluronan binding domain of human CD-44, EGFR, AKR1D1, and HER-2. The best inhibitor among the here-mentioned substituted SOXs was subjected to molecular docking using Autodock 4.2 for the assessment of the interacting residues [73,87]. The grid box for CD-44, EGFR, AKR1D1, and HER-2 was 60 × 60 × 60 points (at x, y, and z, respectively), while the grid spacings for CD-44, EGFR, AKR1D1, and HER-2 were set at 0.419 Å, 0.442 Å, 0.408 Å, and 0.458 Å, respectively. The grid center for the CD-44 was X = 3.0, Y = 10.099, Z = 5.9, for the EGFR was X = 22.013, Y = 0.252, Z = 52.79; for the AKR1D1 was X = 7.35, Y = −30.145, Z = 28.79 and for the HER-2 was X = 17.098, Y = 16.548, Z = 26.600. The **4a**-protein complexes exhibiting the least ΔG (Kcal/mol) and least K*i* values among 40 runs were designated the most stable [67].

### 3.3. In Vitro Antiproliferative Studies of Substituted SOX (***4a***) in PC3 Cells

#### 3.3.1. Materials

The fetal bovine serum (FBS) and RPMI-1640 media were provided by Thermo-Fisher Scientific Pvt. Ltd., Mumbai, India. 2′,7′-dichlorodihydrofluorescein diacetate (H2DCFDA) was acquired from Invitrogen, Waltham, MA, USA. The 3-(4,5-dimethylthiazol-2-yl)-2,5-diphenyltetrazolium bromide (MTT) dye, doxorubicin (reference standard antiproliferative agent) and the solvents dimethyl sulphoxide (DMSO) and isopropanol were procured from Sigma-Merck Ltd., Bangalore, India.

#### 3.3.2. Cell Culture

The human PC-3 cancer cell line was obtained from the cell Repository, NCCS, Pune, India, and was cultured in Gibco™ RPMI-1640 medium (Cat. No. #11875119) consisting of 10% Gibco™ fetal bovine serum (FBS; Cat. No. #A4766801) and 1% Gibco™ antibiotic and antimycotic (Ab/Am) (Cat. No. #15240062) and incubated at 37 °C and 5% CO_2_ [66].

#### 3.3.3. Investigations on the Cytotoxic Effects of Substituted SOX (**4a**) on PC-3 Cells through MTT Assay

The above-screened substituted SOX derivative (**4a**) was evaluated for its anticancer potency together with reference standard doxorubicin against prostate-specific PC-3 cancer cells by the MTT method [66]. The PC-3 cells in RPMI-1640 medium were seeded in a 96-well plate at a seeding density of 5 × 10^3^ cells/well and left overnight at 5% CO_2_ for adherence at 37 °C. After 24 h, varying concentrations (0, 25, 50, 100, 200, and 300 μM) of substituted SOX (**4a**) were added to these adhered PC-3 cells and incubated for a further 24 h in similar atmospheric conditions. The SOX **4a** was first dissolved in DMSO and then serially diluted in the cell culture media (RPMI-1640) to obtain different concentrations ensuring that the final concentration of the DMSO should not exceed 0.5%. After that, 0.2 mL of MTT (20% *v/v*, prepared in RPMI-1640 medium) was added to these cells, incubated for 4 h at 37 °C in the dark, and the generation of formazan crystals was assessed after solubilizing them into the 0.15 mL DMSO by putting the plate on a shaker, and the plate was read at 570 nm.

#### 3.3.4. Assessment of the Impact of the Substituted SOX (**4a**) on the Morphological Features of PC-3 Cells 

The morphological examination of the PC- cells, either in the presence or absence of **4a**, was performed in order to assess the antiproliferative effects of the substituted SOX (**4a**). In brief, the PC-3 cells (10^4^ cells/well) were seeded and incubated with varying doses of **4a** (0, 6.25, 12.5, 25, 50, and 100 μM) at 5% CO_2_ for adherence at 37 °C. Following the incubation period, a FLoidTM Imaging Station, Thermo Fisher Scientific, Waltham, MA, USA, was implied to spot any morphological alterations in the cells.

#### 3.3.5. Detection of ROS Generation

The efficacy of the substituted SOX (**4a**) in prompting ROS production within PC-3 cells was evaluated using DCFH-DA as described recently [66]. In brief, PC-3 cells (10^4^ cells/well) seeded in each well were treated with 0, 6.25, 12.5, 25, 50, and 100 μM substituted SOX (**4a**) for 24 h at 37 °C. The cells were then stained with 10 µM DCFH-DA and incubated for half an hour, and imaging was done using a FLoid imaging station (Thermo-Scientific, Waltham, MA, USA).

#### 3.3.6. DAPI Staining

The ability of substituted SOX (4a) to prompt apoptosis in PC-3 cells was done through DAPI staining [83]. The PC-3 cells were challenged with varying doses of 4a (0, 6.25, 12.5, 25, 50, and 100 μM and incubated for 24 h at 37 °C and then stained with DAPI. DAPI-specific fluorescence in **4a**-treated and untreated cells was captured using a blue filter in a FLoid Imaging station (Thermo-Scientific, Waltham, MA, USA).

## 4. Conclusions

These SOXs (**4a**–**h**) exhibit suitable drug-like features and ADME indices. Moreover, molecular modeling studies using PyRx revealed that 4a, among all SOXs, was found to be the best inhibitor of distinct biomarkers associated with cancer progression, i.e., human CD-44, EGFR, AKR1D1, and HER-2. The enzyme inhibitory activity assessed through AutoDock confirmed that 4a exhibits substantial binding affinity for CD-44, EGFR, AKR1D1, and HER-2 (∆G: −6.65, −6.55, −8.73, and −7.27 Kcal/mol, respectively), which were also comparable to the standard doxorubicin. Apart from the in-silico analysis, the **4a** also greatly affected the morphological features of the PC-3 cells. Moreover, the DCFH-DA and DAPI staining also confirmed the antiproliferative potential of 4a via substantial ROS generation as well as nuclear condensation into the PC-3 cells. This initial in silico and in vitro report demonstrate substituted SOX (**4a**) as a potent chemotherapeutic agent via its ability to interfere with the human CD-44, EGFR, AKR1D1, and HER-2 as well as instigating the ROS generation and nuclear condensation in PC-3 cells. 

## Figures and Tables

**Table 1:** Conditions for the reaction optimization.

Entry	*p*-TSA (mM)	Solvent	Time (h)	Temp.	Yield (%)
1	0	EtOH	16	RT	trace
2	0.25	EtOH	12	RT	55
3	0.25	EtOH	10	RT	55
4	0.5	EtOH	10	RT	62
5	0.5	EtOH	6	RT	70
6	0.5	EtOH	8	RT	95
7	0.7	EtOH	10	RT	60
8	0.7	EtOH	8	RT	65
9	0.5	MeOH	8	RT	65
10	0.5	EtOH:H_2_O (2:1)	8	RT	trace
11	0.8	EtOH: H_2_O (2:1)	8	RT	trace
12	0.5	EtOAc	8	RT	ND
13	0.5	Acetone	8	RT	ND
14	0.5	Dime. sulfoxide	8	RT	ND
15	0.5	Isopropanol	8	RT	trace

Reaction condition: The reaction (10 mL) was carried out with isatin (1 mM), dimethyl acetylenedicarboxylate (1 mM), 4-chloro 3-(trifluoromethyl) aniline (1 mM), and (0.5 mM) *p*-TSA acid catalyst. The isolated yield was calculated. Reaction completion was monitored by TLC. RT = room temperature. ND = not detected.

**Table 2:** Synthesis of SOXs (**4a**–**h**) from substituted DOX (**1a-c**), dimethyl acetylenedicarboxylate 3, and aryl amine (**2a**-**c**).

Entry	DOX	Arylamine	Time (h)	SOX	Melting Point (°C)	Yield (%)
1	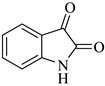 **1a**	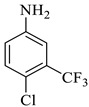 **2a**	8	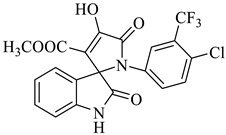 **4a**	244	95
2	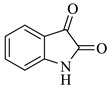 **1a**	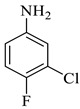 **2b**	8	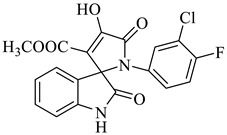 **4b**	243	79
3	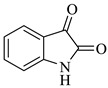 **1a**	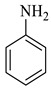 **2c**	6	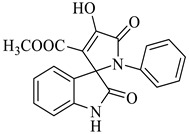 **4c**	245	83
4	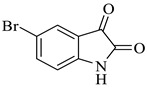 **1b**	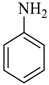 **2c**	9	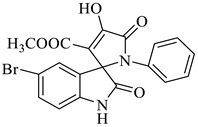 **4d**	248	84
5	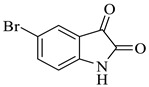 **1b**	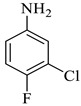 **2b**	12	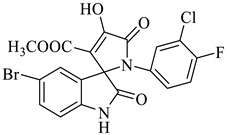 **4e**	239	74
6	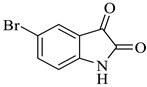 **1b**	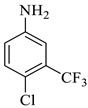 **2a**	8	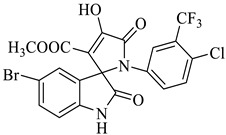 **4f**	236	81
7	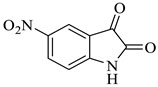 **1c**	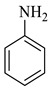 **2c**	8	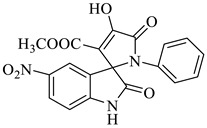 **4g**	246	84
8	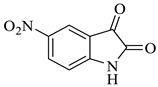 **1c**	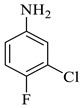 **2b**	8	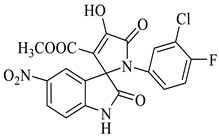 **4h**	241	95

**Table 3:** PyRx-based screening of substituted SOXs (**4a**–**h**) against CD44, EGFR- Tyrosine kinase domain, 5-β-reductase (AKR1D1), and HER-2.

S. No.	Compounds	CD44 (PDB ID: 1UUH)	EGFR (PDB ID: 1M17)	AKR1D1 (PDB ID: 3CQA)	HER-2 (PDB ID: 3PP0)
1	**4a**	**−7.4**	**−8.7**	**−10.1**	**−7.8**
2	**4b**	−7.0	−8.3	−9.3	−6.8
3	**4c**	−7.1	−8.0	−9.4	−6.6
4	**4d**	−7.2	−8.2	−8.1	−6.8
5	**4e**	−6.7	−8.3	−7.6	−7.1
6	**4f**	−7.2	−8.2	−7.1	−7.4
7	**4g**	−6.5	−8.0	−9.9	−7.7
8	**4h**	−6.8	−8.4	−9.8	−7.3
9	* Doxorubicin	−7.0	−10.1	−10.4	−7.7

* Reference standard drug.

**Table 4:** Deep analysis of the **4a** binding affinities and interacting residues of human CD44, EGFR- Tyrosine kinase domain, 5-β-reductase (AKR1D1), and HER-2.

Complex	Binding Energy	Interacting Residues
EGFR-4a	−6.55 Kcal/mol	Leu694, Val702, Ala720, Lys721, Leu768, Met769, Gly772, Cys773, Arg817, Asn818, Leu820, Thr830, Asp831
EGFR-Doxo	−10.11 Kcal/mol	Leu694, Phe699, Ala719, Lys721, Glu738, Met742, Val762, Leu764, Thr766, Gln767, Leu768, Met769, Pro770, Gly772, Arg817, Asn818, Leu820, Thr830, Asp831, Phe832
CD44-4a	−6.65 Kcal/mol	Arg29, Phe34, Phe56, Asn120, Thr130, Ser131, Val132, Thr133, Asp134, Pro136, Ser158
CD44-Doxo	−5.83 Kcal/mol	Arg29, Asn57, Asn120, Ala121, Ser122, Ala123, Pro124, Asn128, Thr130, Ser131, Val132, Thr133, Asp134, Leu135, Pro136
AKR1D1-4a	−8.73 Kcal/mol	Gly24, Thr25, Tyr26, Ser27, Glu28, Tyr58, Lys87, Tyr219, Ser220, Pro221, Ser225, Tyr230, Ile271, Pro272, Gly273, Val309
AKR1D1-Doxo	−10.01 Kcal/mol	Gly24, Tyr26, Ile57, Tyr58, Lys87, Trp89, Val121, Glu120, Tyr132, Asn170, Gln193, Tyr219, Ser220, Pro221, Thr224, Trp230, Ile271, Lys273, Val309, Leu311, Met313, Trp314, Phe322
HER-2-4a	−7.27 Kcal/mol	Gly77, Ser78, Lue726, Gyl729, Val734, Ala751, Leu800, Met801, Gly804, Cys805, Asp808, Asp850, Leu852, Arg859, Thr862, Asp863
HER-2-Doxo	−8.94 Kcal/mol	Lys724, Val725, Leu726, Val734, Ala751, Thr798, Leu800, Met801, Pro802, Tyr803, Gly804, Cys805, Asp808, Arg849, Asn850, Leu852, Thr862

## Data Availability

Not applicable.

## Supplementary Materials

The supporting information can be downloaded at: https://www.mdpi.com/article/10.3390/ijms24087336/s1.
