# Supplementary material for: Novel Functionalized Spiro [Indoline-3,5′-pyrroline]-2,2′dione Derivatives: Synthesis, Characterization, Drug-Likeness, ADME, and Anticancer Potential
Source: Int J Mol Sci. 2023 Apr 15;24(8):7336. doi: 10.3390/ijms24087336 (PMC10139052; doi:10.3390/ijms24087336)
Supplement: Supplementary file 1 [file ijms-24-07336-s001.zip › ijms-2117110-supplementary.pdf]

Supplementary Material

Novel functionalized spiro [indoline-3, 5'-pyrroline]-2, 2'dione derivatives: Synthesis, characterization, drug-likeness, ADME, and anti-cancer potential

Mohd Asif[1], Sahir Sultan Alvi[2], Tazeen Azaz [3], Abdul Rahman Khan [1], Bhoopendra Tiwari [3], Bilal Bin Hafeez [2,*] and Malik Nasibullah[1,*]

[1] Department of Chemistry, Integral University, Lucknow, 226026, U.P., India.; machem86@gmail.com (M.A.); arahman@iul.ac.in (A.R.K.)
[2] Department of Immunology and Microbiology, South Texas Center of Excellence in Cancer Research, School of Medicine, University of Texas Rio Grande Valley, McAllen, 78504, Texas (TX), USA; sahir.alvi@utrgv.edu (S.S.A.)
[3] Department of Biological and Synthetic Chemistry, Centre of Biomedical Research, SGPGIMS-Campus, Raebareli Road, Lucknow226014, U.P., India; tazeen0@gmail.com (T.A.); btiwari@cbmr.res.in (B.T.)
* Correspondence: malik7860@gmail.com (M.N.); Tel.: M. N.: +91-9919695886 (M.N.); bilal.hafeez@utrgv.edu (B.B.H.); Tel.: +1(901)-608-2182 (B. B. H.)

Materials and Methods

The reaction was initiated by mixing isatin(1mM), 4-chloro-3-(trifluoromethyl)aniline (1mM), and dimethyl acetylenedicarboxylate (1mM) in 5 mL of different solvents (*i.e.*, ethanol, MeOH, EtOAc, acetone, DMSO, and isopropanol) in the presence of *p*-TSA (0.0, 0.25, 0.50 and 0.75 mM; catalyst) followed by stirring the mixture at room temperature for varying durations (6-16 h) (Scheme 1). Following the designated optimization steps with above-mentioned three components, the concentrated precipitates were collected and washed with respective solvent in order to obtain pure compounds devoid of *p*-TSA. On the basis of the findings from these optimizations hits, we concluded that the optimal conditions for the synthesis of the novel spirooxindole pyrrolines (4a) were;*p*-TSA: 0.5 mM and solvent: EtOH. The rest of the compounds (4b-h) were synthesized by using distinct isatin derivatives, aryl amine derivatives and dimethyl acetylenedicarboxylate (remained constant) in presence of 0.5 mM *p*-TSA and ethanol at RT.

Apparatus used for the characterization

The melting point of all the synthesized compounds wasdetermined with the help of open capillary tube method using IA 9100 MK-Digital uncorrected melting Point analyzer Griffin Apparatus at Department of Chemistry, Integral University. Infrared Spectroscopic analysis was done on Agilent Cary 630 FT-IR Spectrometer (Range: 4000-450 cm^{-1}) Perkin-Elmer Spectrum version 10.03.06 at CSIR-CDRI, Lucknow and the spectra were expressed as wave number (cm^{-1}) with KBr discs. ^{1}H NMR Spectra wasdetermined using Advance-400 MHz Bruker, Switzerland and Bruker AVLL-300 MHz (Bruker, Switzerland) using DMSO-d_6 as a solvent. The chemical shifts were denoted by δ ppm units using trimethylsilane as the internal standard. ^{13}C NMR spectra were recorded using Bruker AVLL-100 MHz (Bruker, Switzerland) using DMSO-d_6. High Resolution Mass Spectra was taken on Acquisition SW-6200 series TOF, version Q-TOF B.05.00 at PGI-Lucknow. The completion of all the reactions was monitored with the helpof thin layer chromatography (TLC) and using variable proportions of ethyl acetate and petroleum n-hexane.On the contrary, the purity of the synthesized compound which was used for in-vitro studieswasassessed through the HPLC at Integral University, Lucknow.Briefly, 20 µl SOX **4a** was analyzed using Shimadzu 20AD Gradient LC System with PDA Detector system. A C-18 column (4.6 mm×250 mm, 5 µm) was used to achieve the chromatographic separation.The mobile phase comprising of methanol: H_2O (70:30, v/v) was applied at a flow rate of 0.5 ml/min. The SOX **4a** was detected at 254 nm with a retention time of 7.928 min in HPLC system thermo-stated at 25 °C.

HPLC data of methyl-1'-(4-chloro-3-(trifluoromethyl)phenyl)-4'-hydroxy-2,5'-dioxo-1',5'-dihydrospiro[indoline-3,2'-pyrrole]-3'-carboxylate (4a)

SHIMADZU LabSolutions

Analysis Report

<Sample Information>

Sample Name	: 4A R2		
Sample ID	: Chem		
Data Filename	: 4A R2.lcd		
Method Filename	: WM 1535 T2.lcm		
Batch Filename	:		
Vial #	: 1-1	Sample Type	: Unknown
Injection Volume	: 20 uL		
Date Acquired	: 12/21/2022 2:07:24 PM	Acquired by	: System Administrator
Date Processed	: 12/21/2022 2:42:27 PM	Processed by	: System Administrator

<Chromatogram>

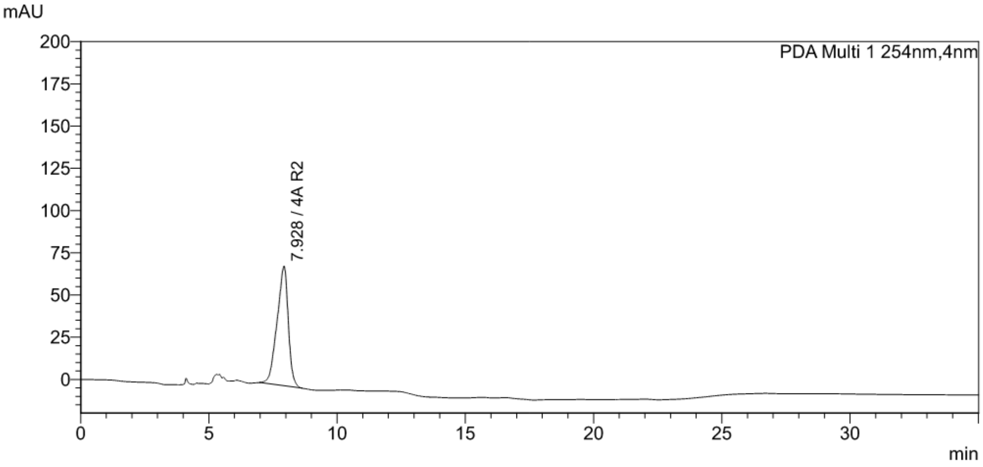

<Peak Table>

PDA Ch1 254nm

Peak#	Ret. Time	Area	Height	Conc.	Unit	Mark	Name
1	7.928	2157752	70910	0.000	mg/L	M	4A R2
Total		2157752	70910				

USP Width	Lambda max	Noise	Tailing Factor
0.821	254/328/199/218/425	225.09	0.846

Copies of Characterization details (4a-h)

FT-IR Spectra (4a)

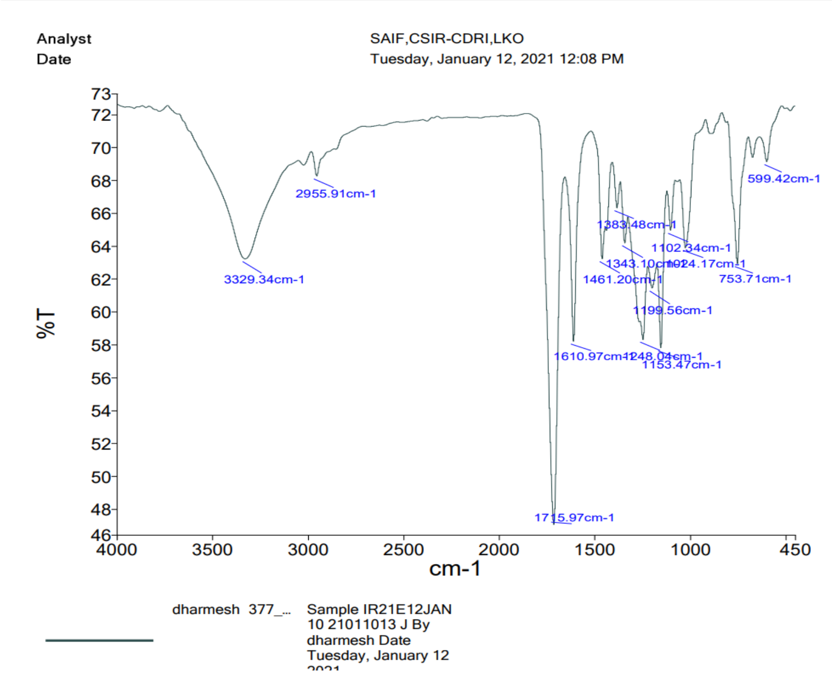

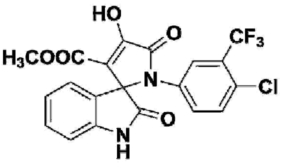

^{13}C NMR Spectra (4a)

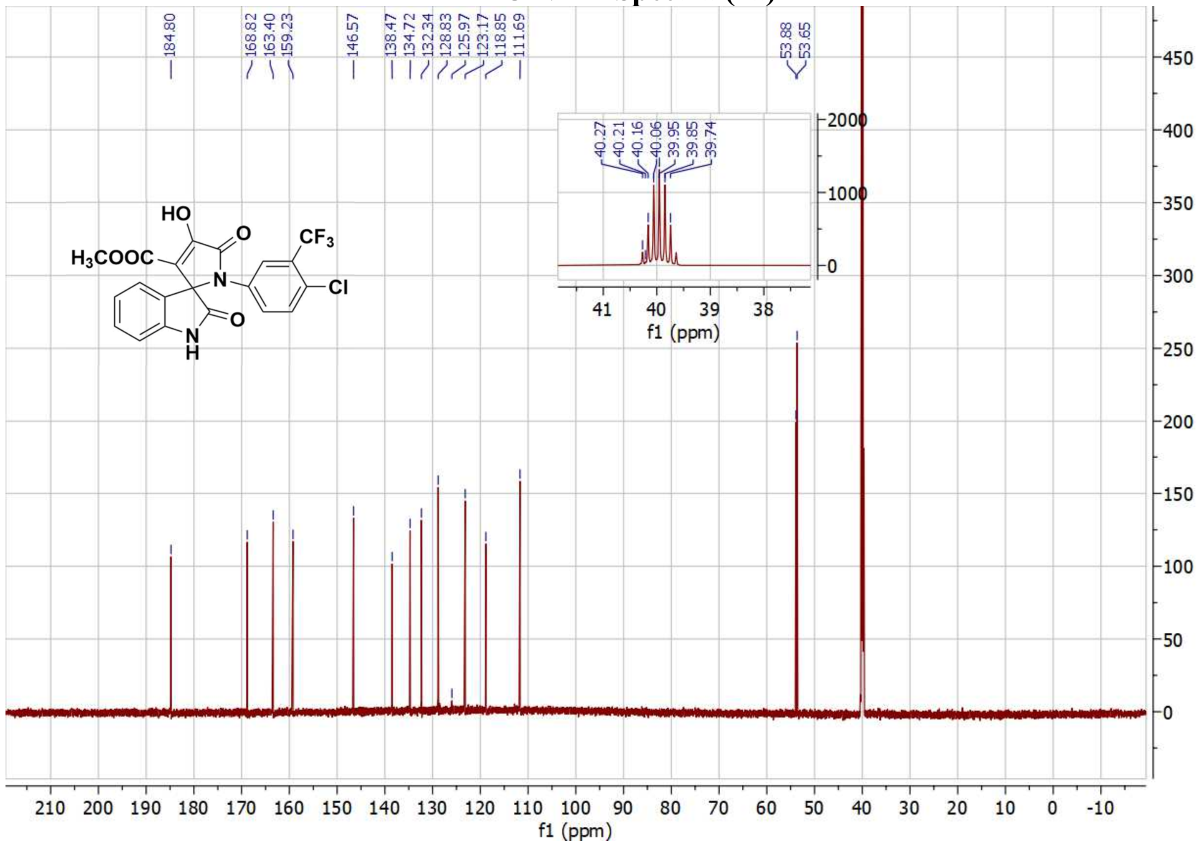

^{1}H NMR Spectra (4a)

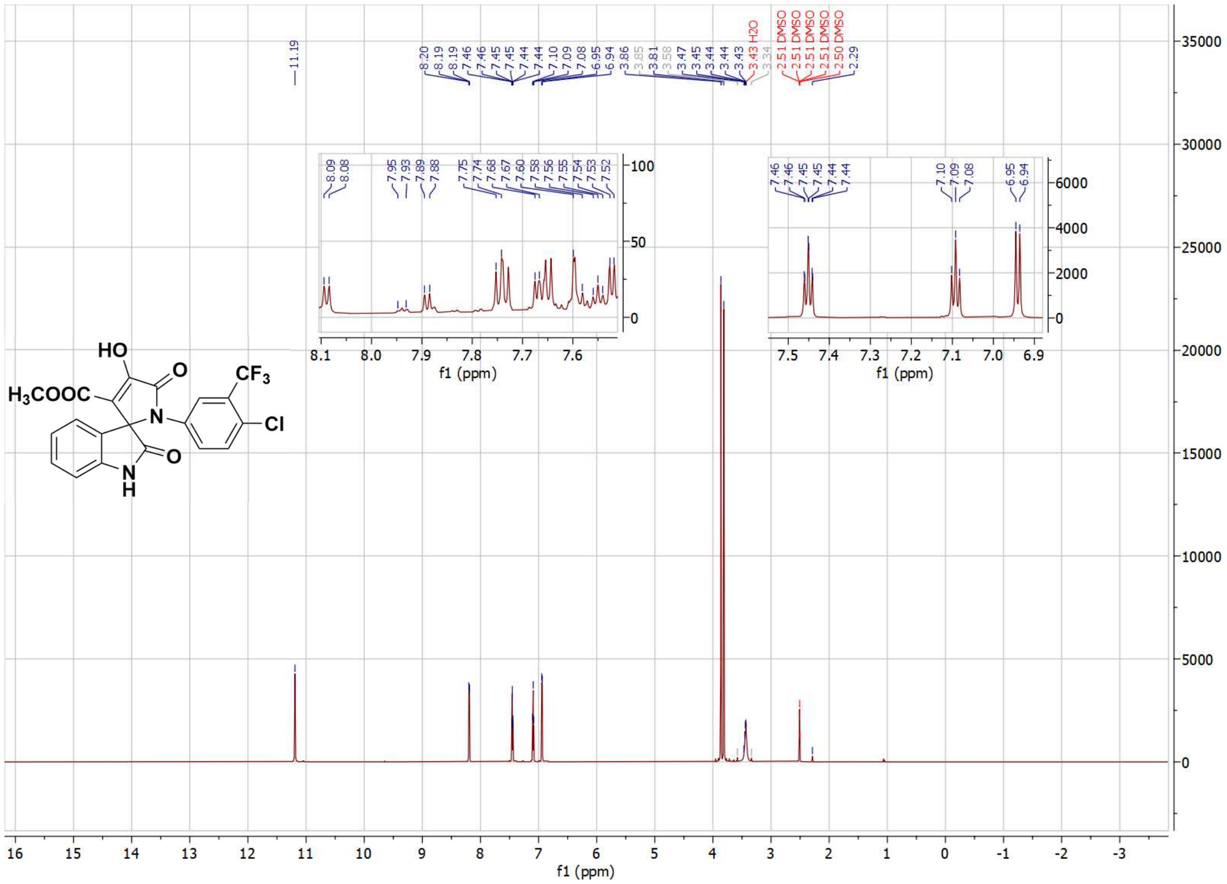

HRMS (4a)

Qualitative Analysis Report

**Data Filename**	TZ-3-J.d	**Sample Name**	TZ-3-J
**Sample Type**	Sample	**Position**	Vial 23
**Instrument Name**	Instrument 1	**User Name**	
**Acq Method**	Direct Mass-2017.m	**Acquired Time**	2/4/2021 10:14:55 PM
**IRM Calibration Status**	Some Ions Missed	**DA Method**	Default.m
**Comment**			

Sample Group **Info.**
Acquisition SW 6200 series TOF/6500 series
Version Q-TOF B.05.00 (B5042.0)

User Chromatograms

Fragmentor Voltage 118 **Collision Energy** 0 **Ionization Mode** ESI

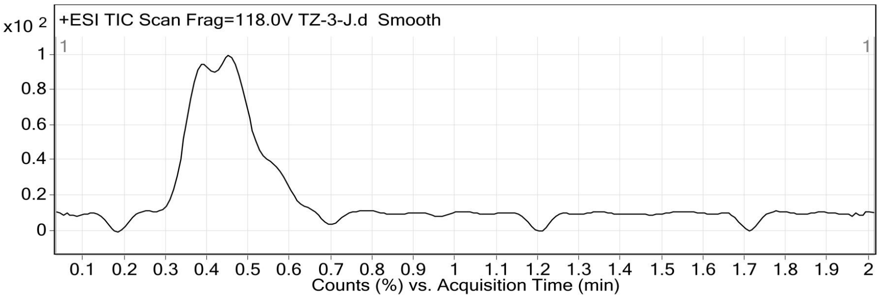

User Spectra

Fragmentor Voltage	Collision Energy	Ionization Mode
118	0	ESI

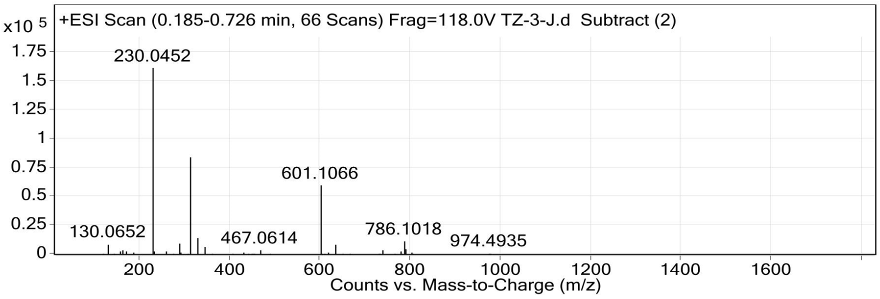

Peak List

m/z	z	Abund
130.0652	1	9179.41
230.0452	1	161993.55
231.0482	1	20138.81
290.0663	1	9690.5
312.0483	1	84380.41
313.0514	1	12547.83

328.0221	1	14118.4
601.1066	1	59855.86
602.1097	1	18067.87
786.1018	1	11524.22

Compounds

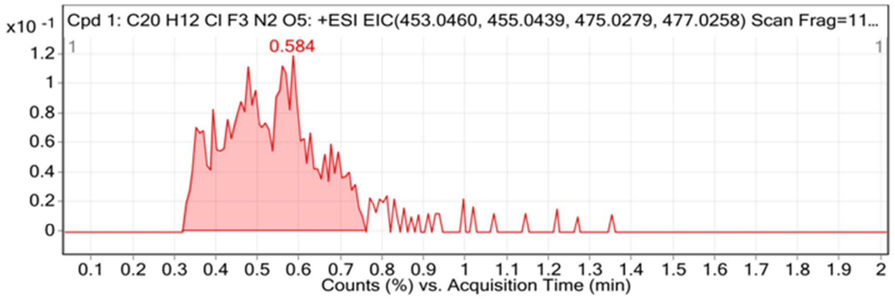

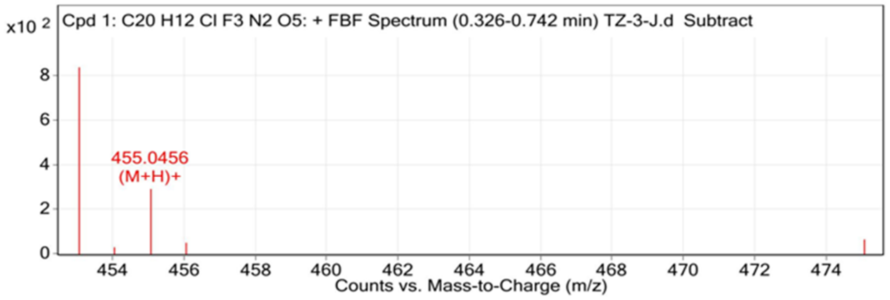

Peak List

m/z	z	Abund	Formula	Ion
453.046	1	842.68	C20H13ClF3N2O5	(M+H)+
454.0439	1	36.8	C20H13ClF3N2O5	(M+H)+
455.0456	1	297.7	C20H13ClF3N2O5	(M+H)+
456.0508	1	55.77	C20H13ClF3N2O5	(M+H)+
475.0304	1	70.14	C20H12ClF3N2NaO5	(M+Na)+

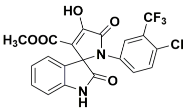

Chemical Formula: $C_{20}H_{12}ClF_3N_2O_5$
Molecular Weight: 453,
HRMS (ESI-TOF m /z) = $(M + H)^+$
Calculated for $C_{20}H_{13}ClF_3N_2O_5^+$ 454.00784,
Found 453.046; 454.0439; 455.0456; 456.0508;
HRMS (ESI-TOF m /z) = $(M + Na)^+$
Calculated for $C_{20}H_{12}ClF_3N_2NaO_5^+$ 475.9897,
Found 475.0304.

FT-IR Spectra (4b)

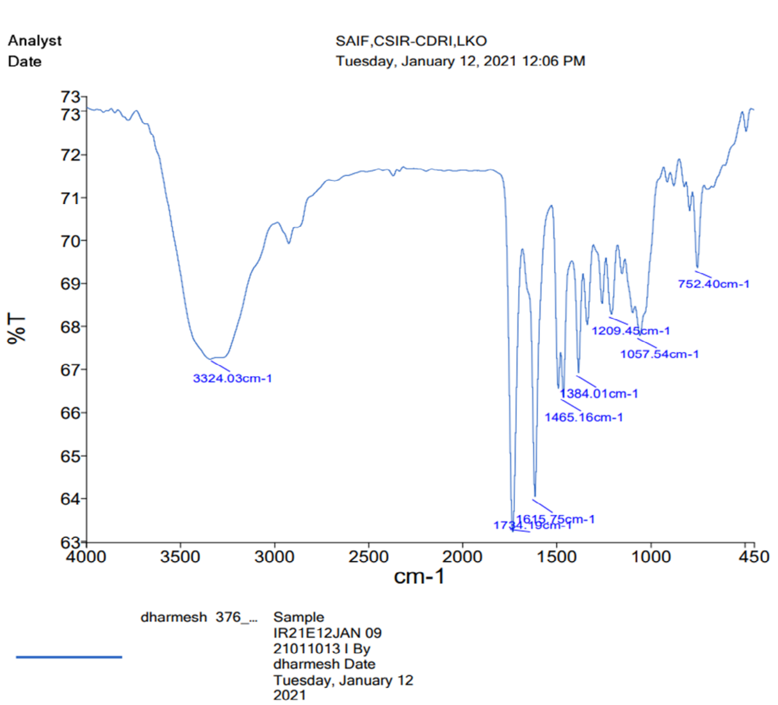

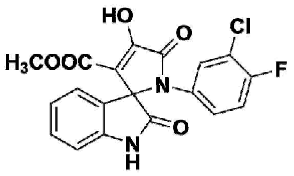

^{1}H NMR Spectra (4b)

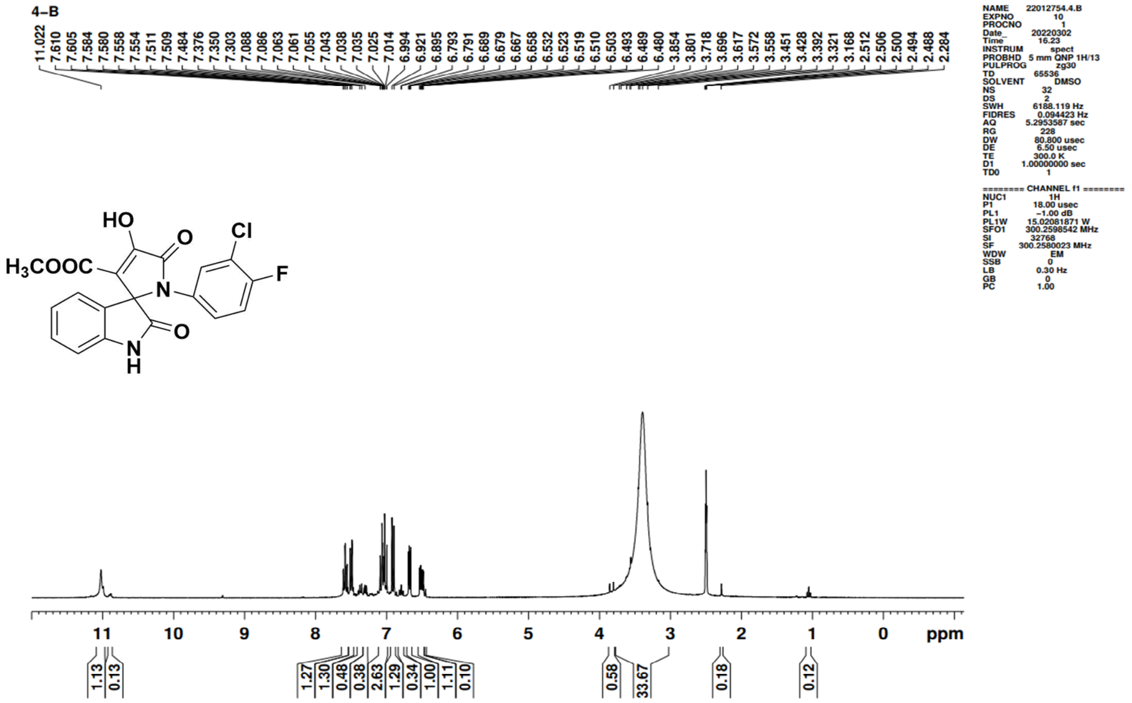

^{13}C NMR Spectra (4b)

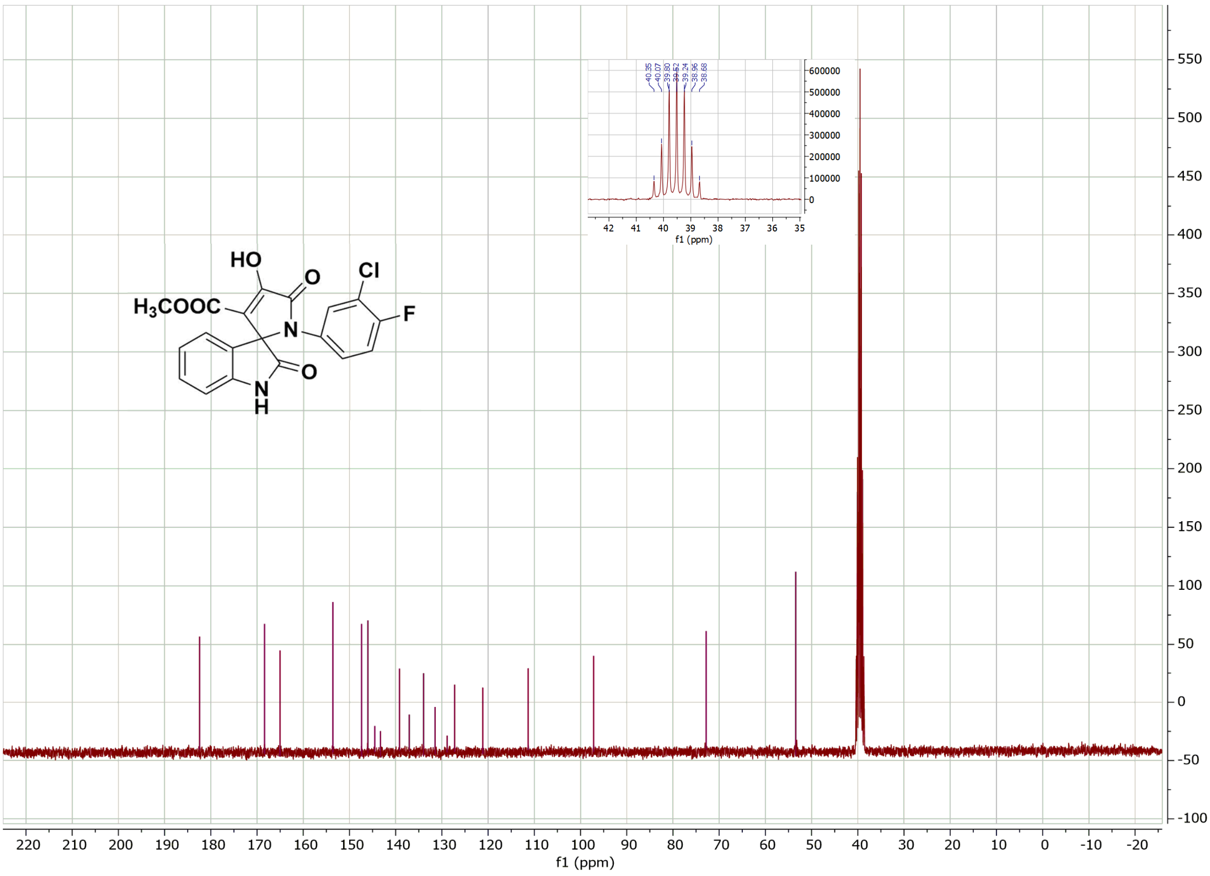

HRMS (4b)

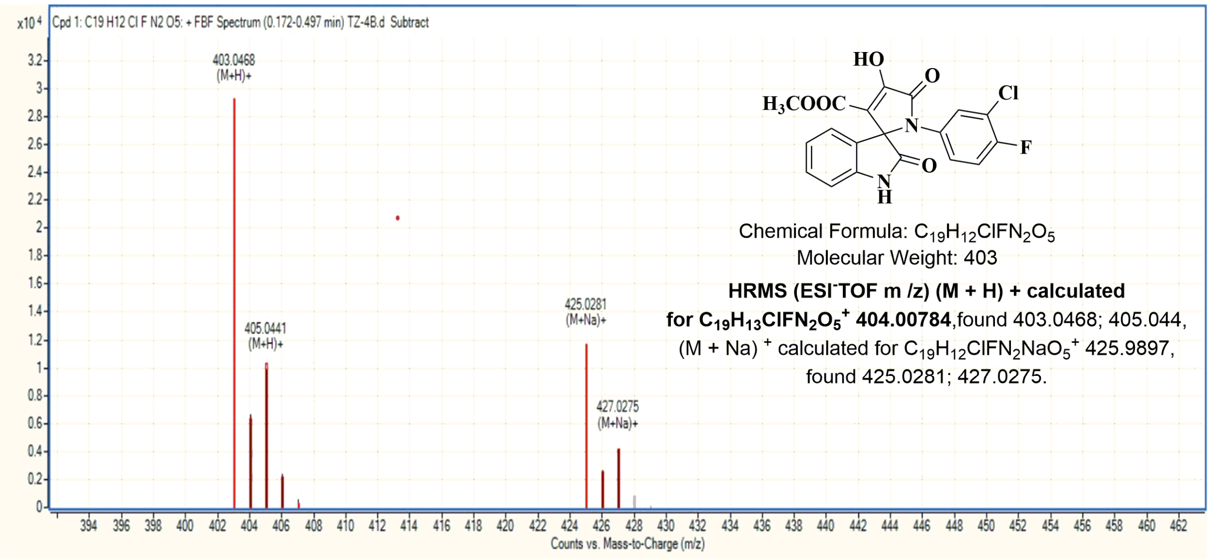

FT-IR Spectra (4c)

PerkinElmer Spectrum Version 10.03.06
Tuesday, January 12, 2021 12:04 PM

Analyst SAIF,CSIR-CDRI,LKO
Date Tuesday, January 12, 2021 12:04 PM

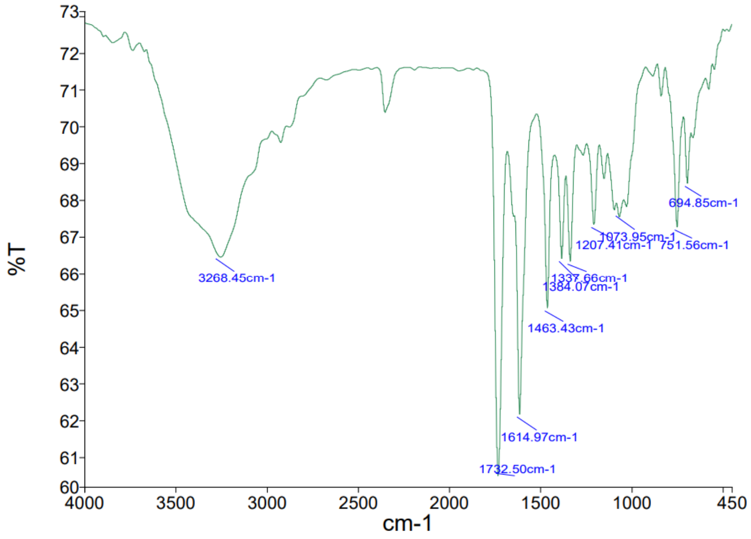

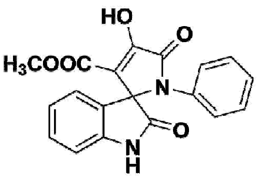

dharmesh 375_... Sample IR21E12JAN 08 21011013 H By dharmesh Date Tuesday, January 12 2021

^{1}H NMR Spectra (4c)

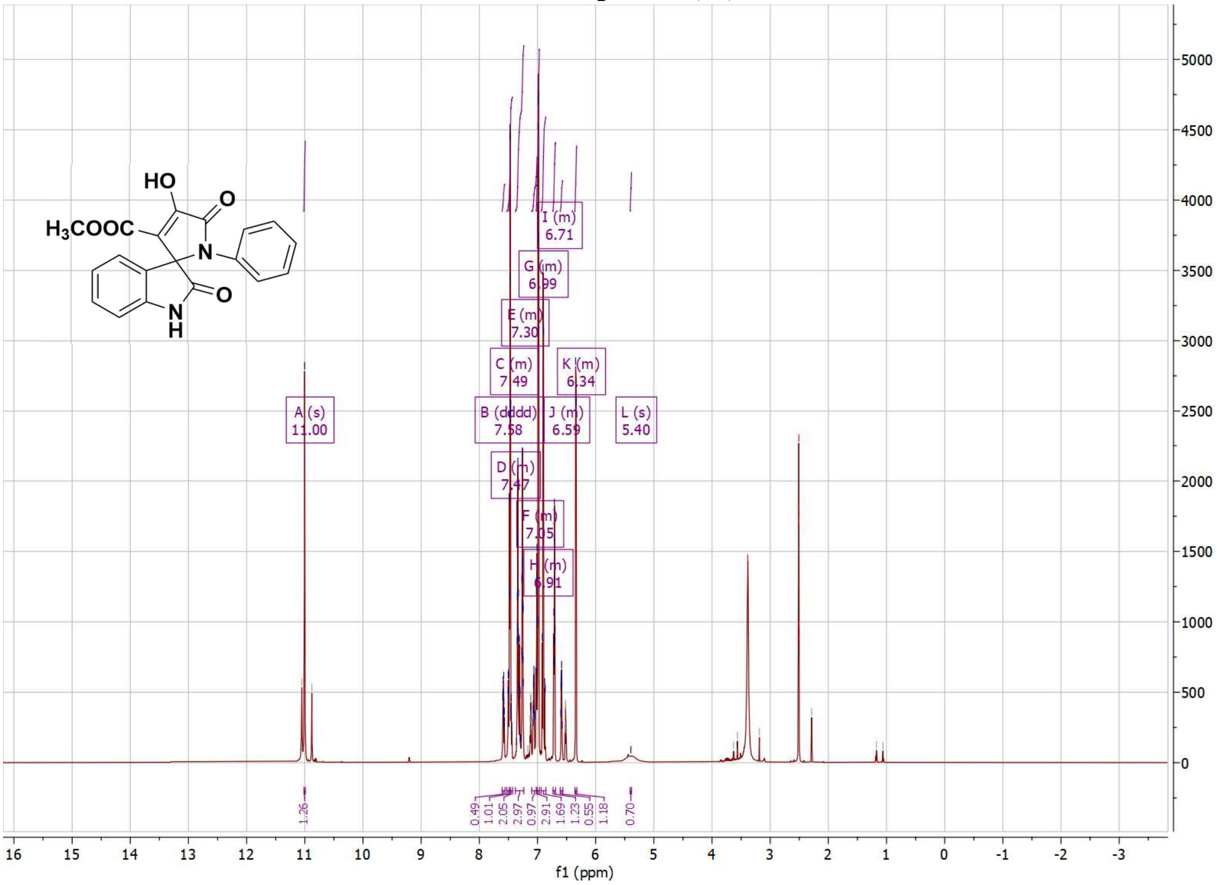

^{13}C NMR Spectra (4c)

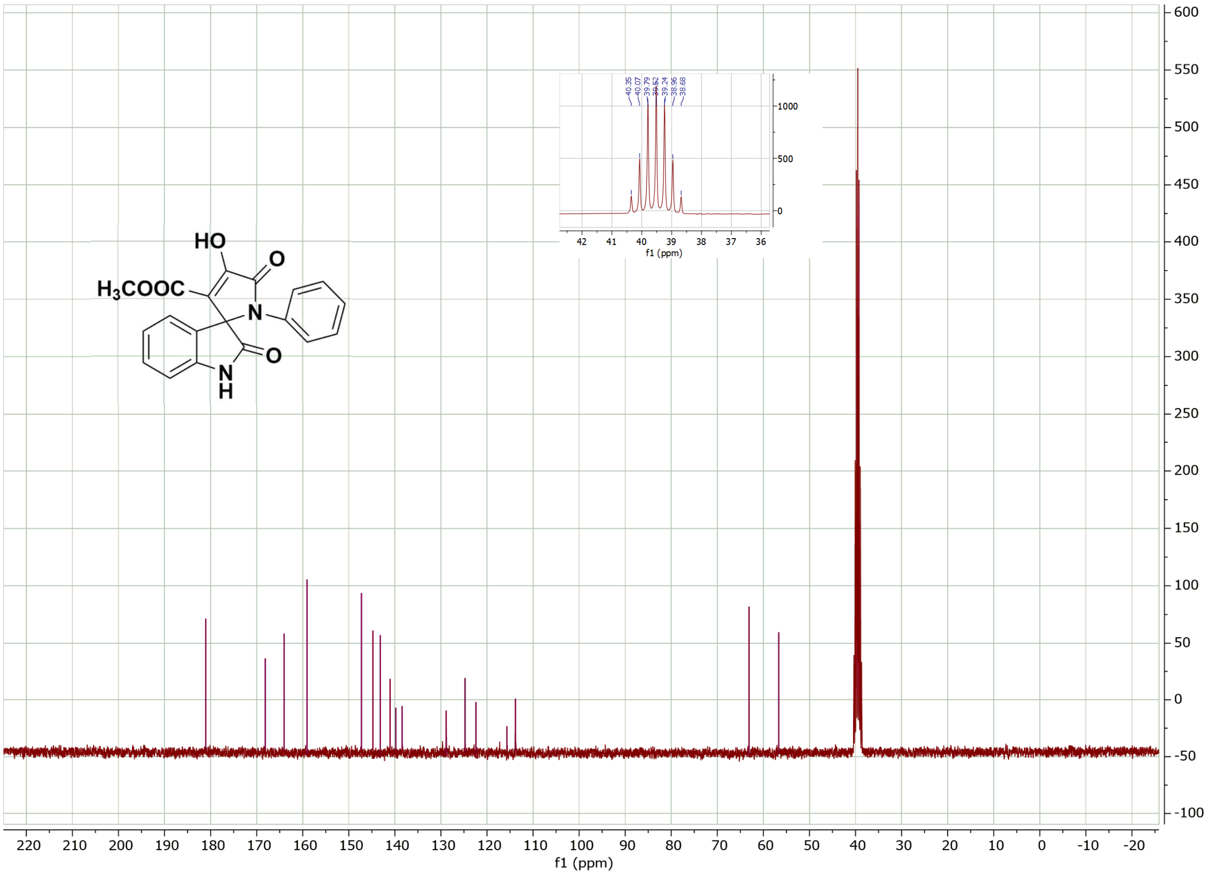

FT-IR Spectra (4d)

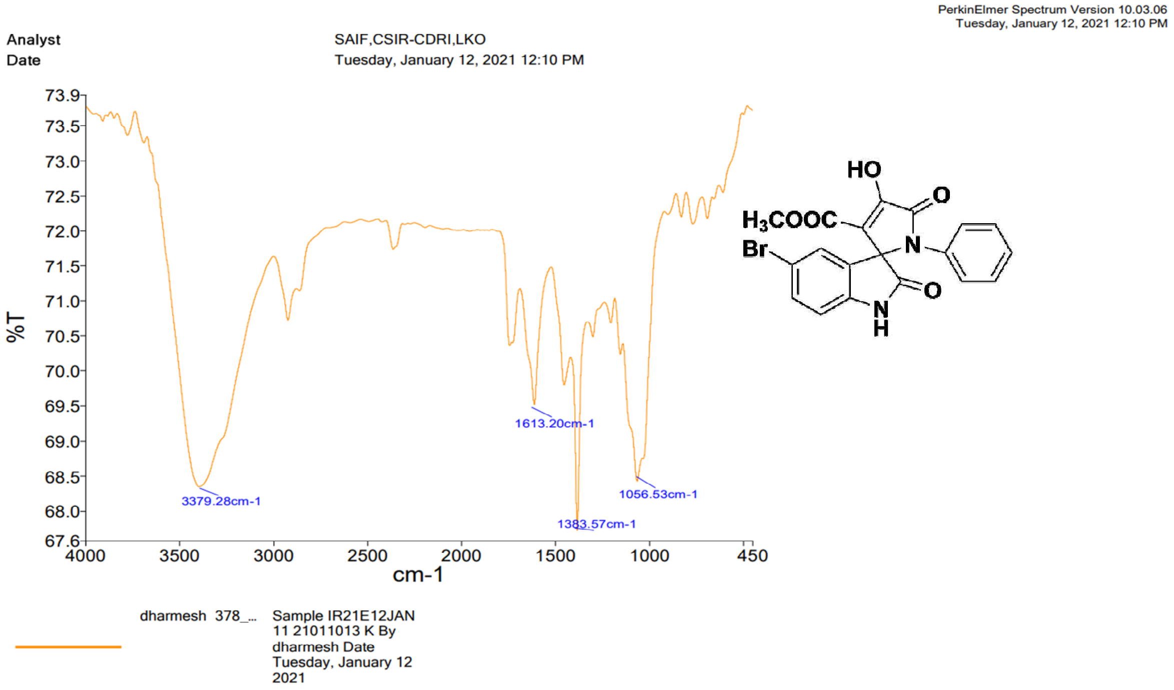

^{1}H NMR Spectra (4d)

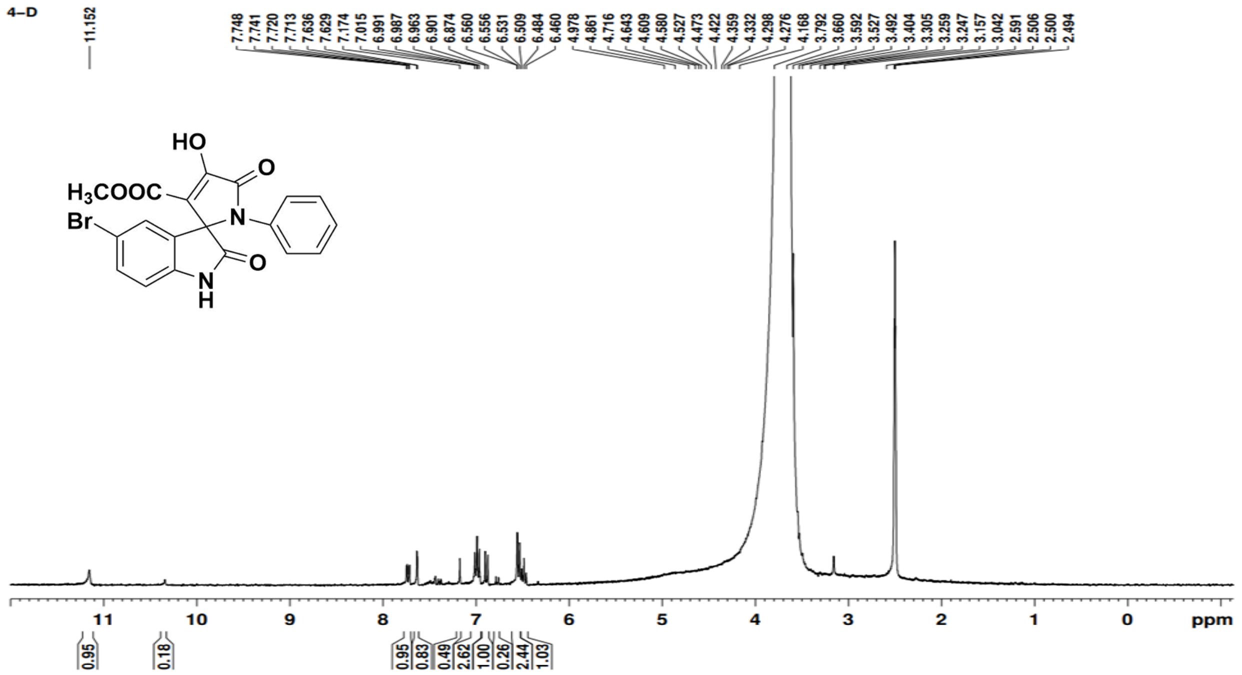

^{13}C NMR Spectra (4d)

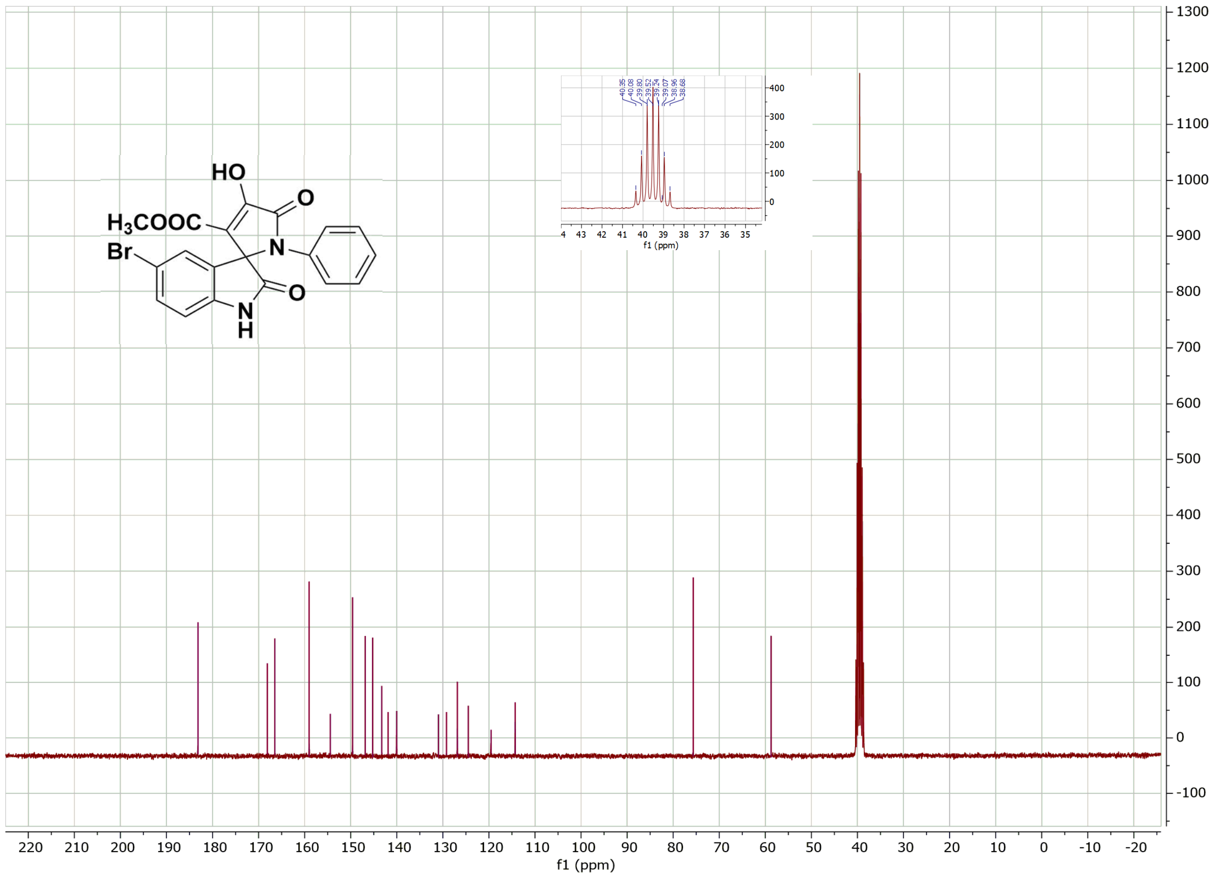

FT-IR Spectra (4e)

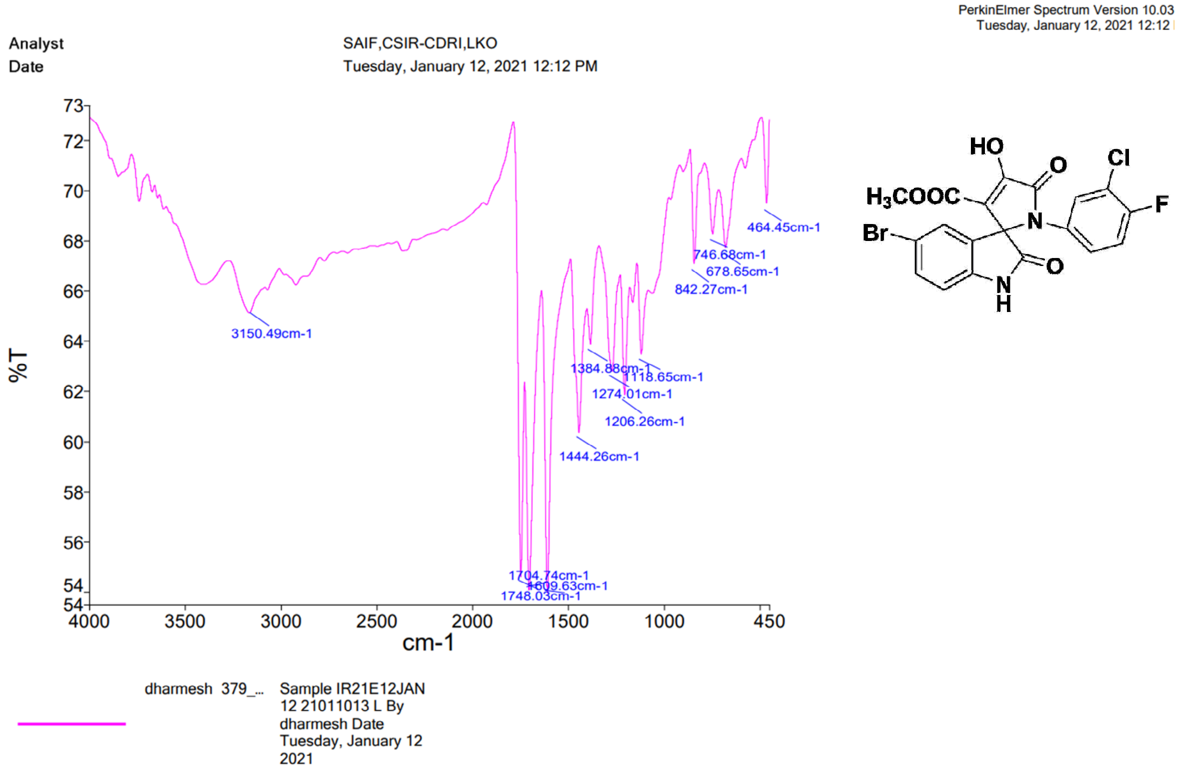

^{1}H NMR Spectra (4e)

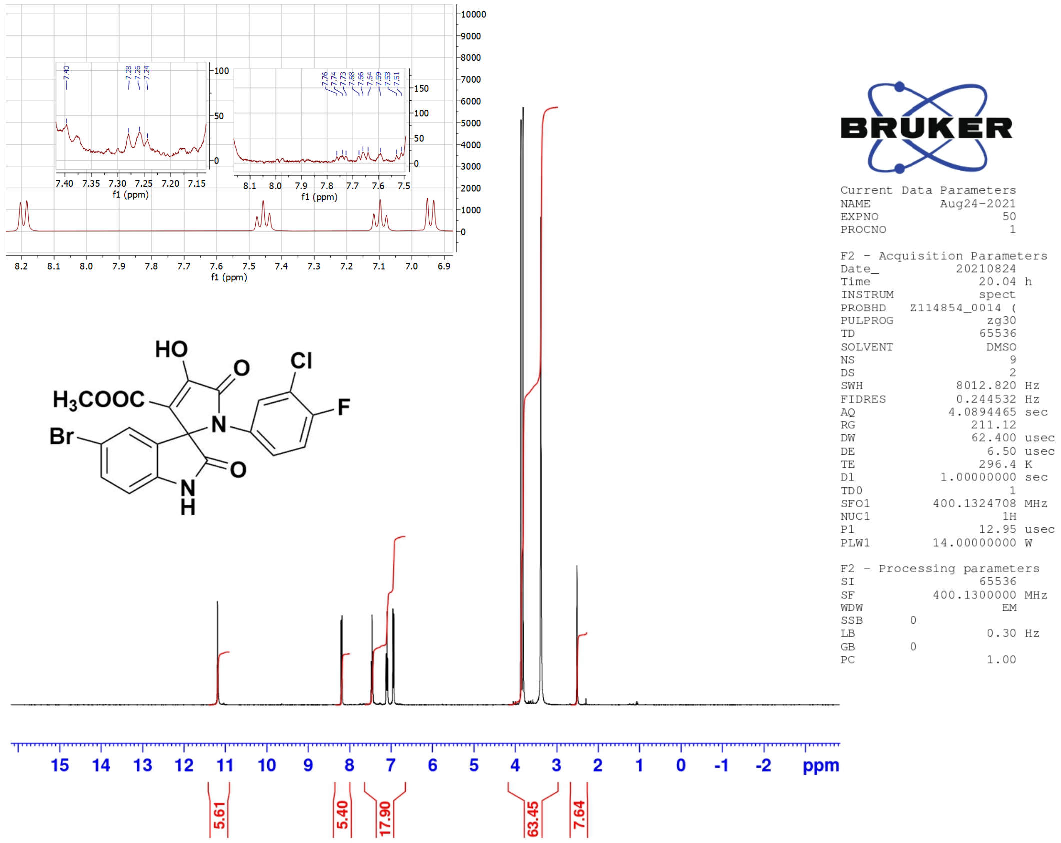

^{13}C NMR Spectra (4e)

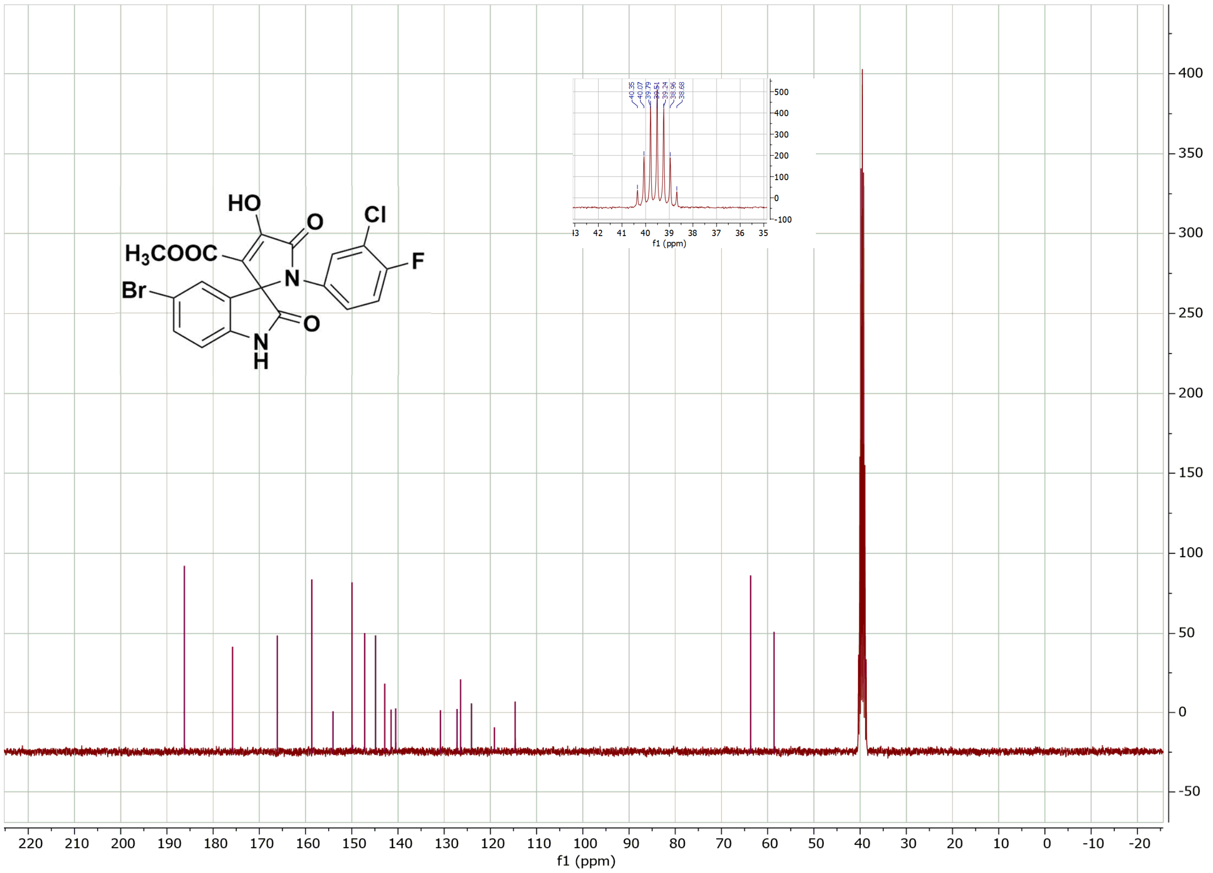

FT-IR Spectra (4f)

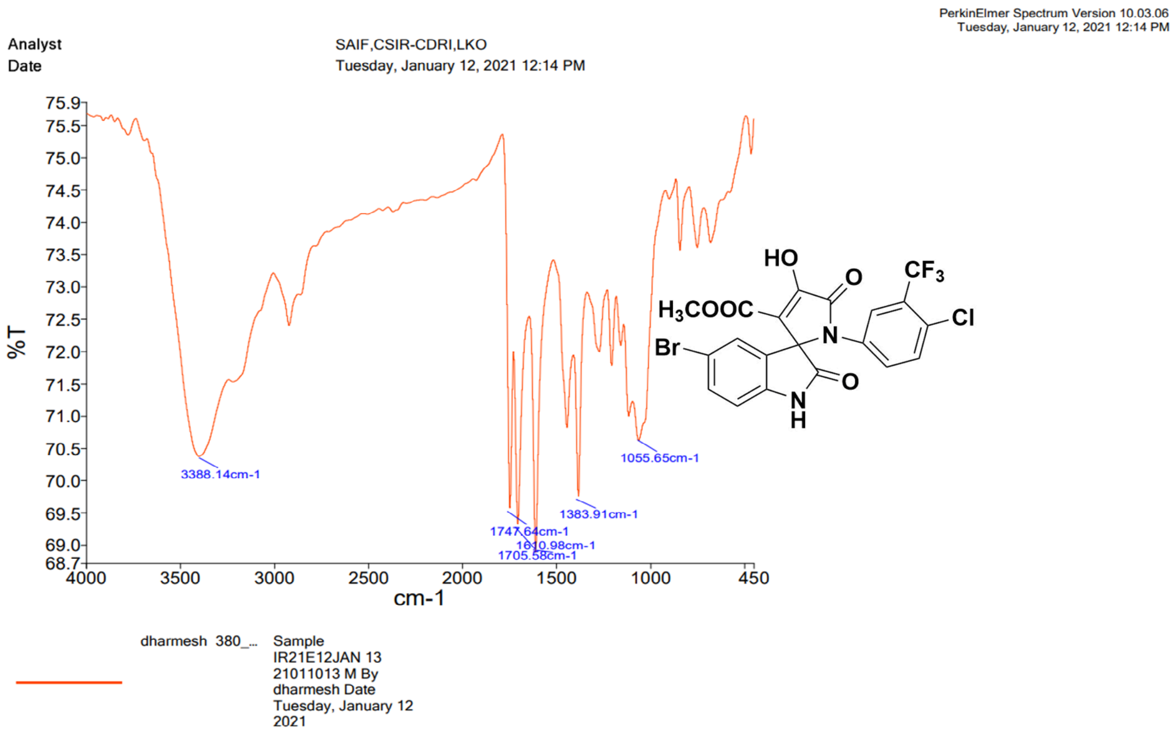

^{13}C NMR Spectra (4f)

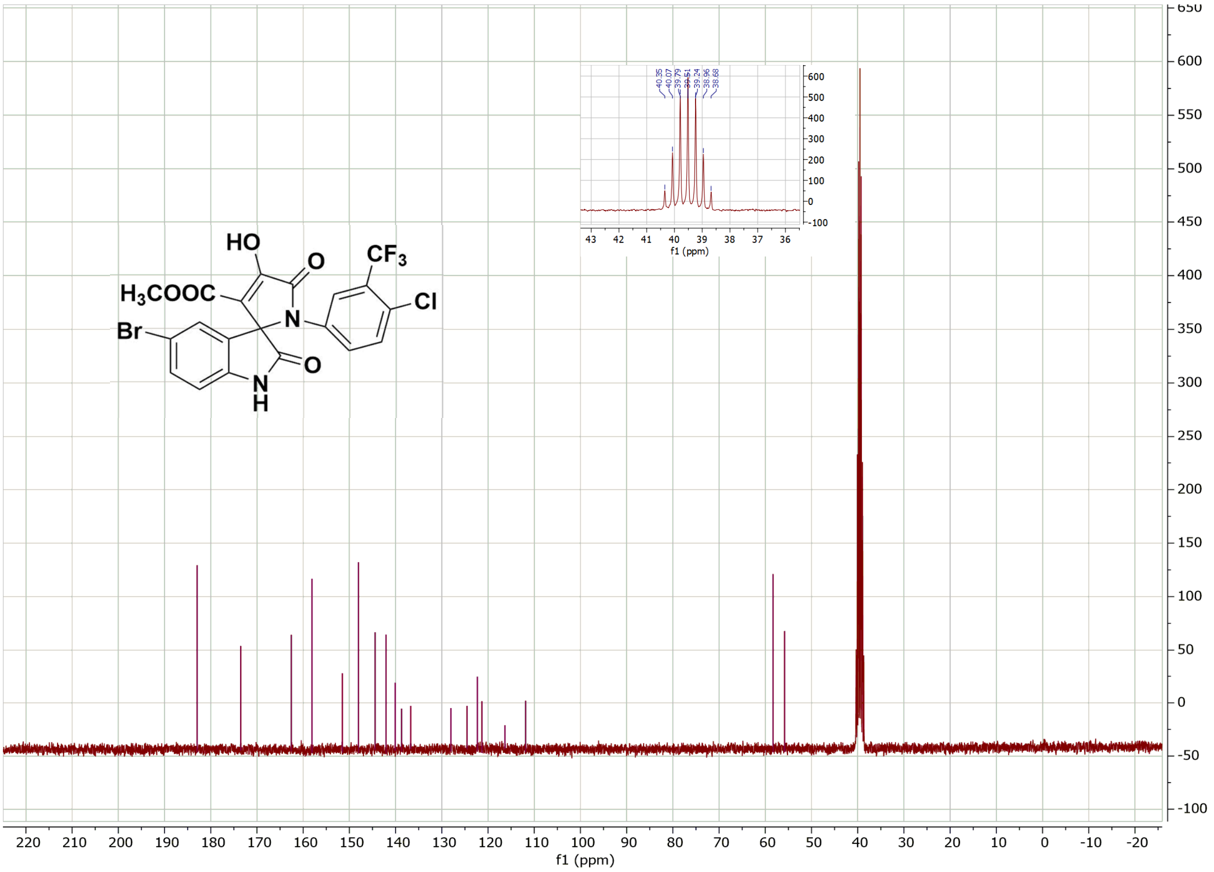

^{1}H NMR Spectra (4f)

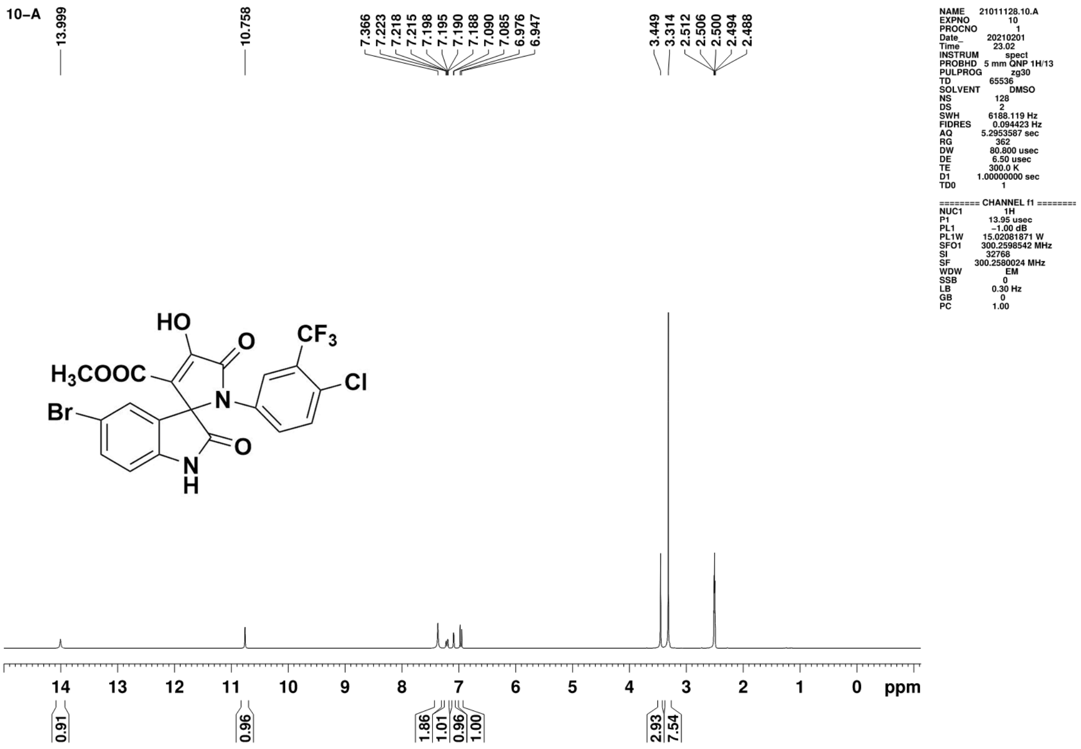

FT-IR Spectra (4g)

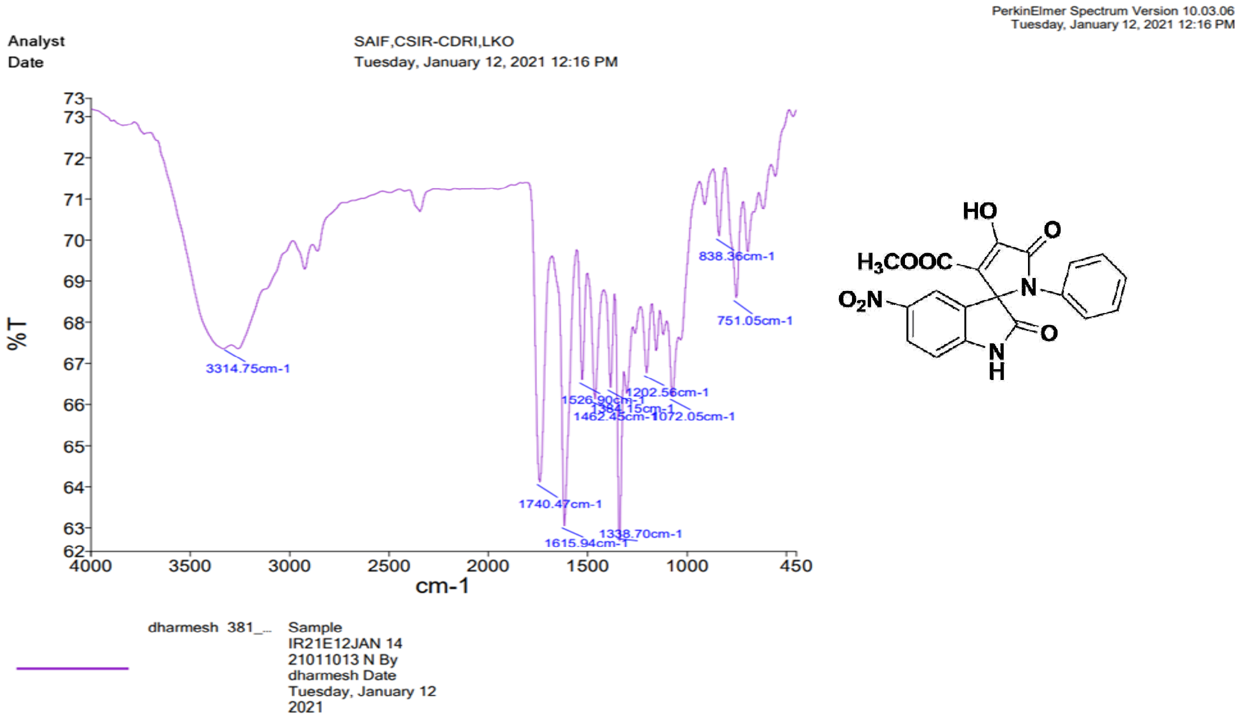

^{13}C NMR Spectra (4g)

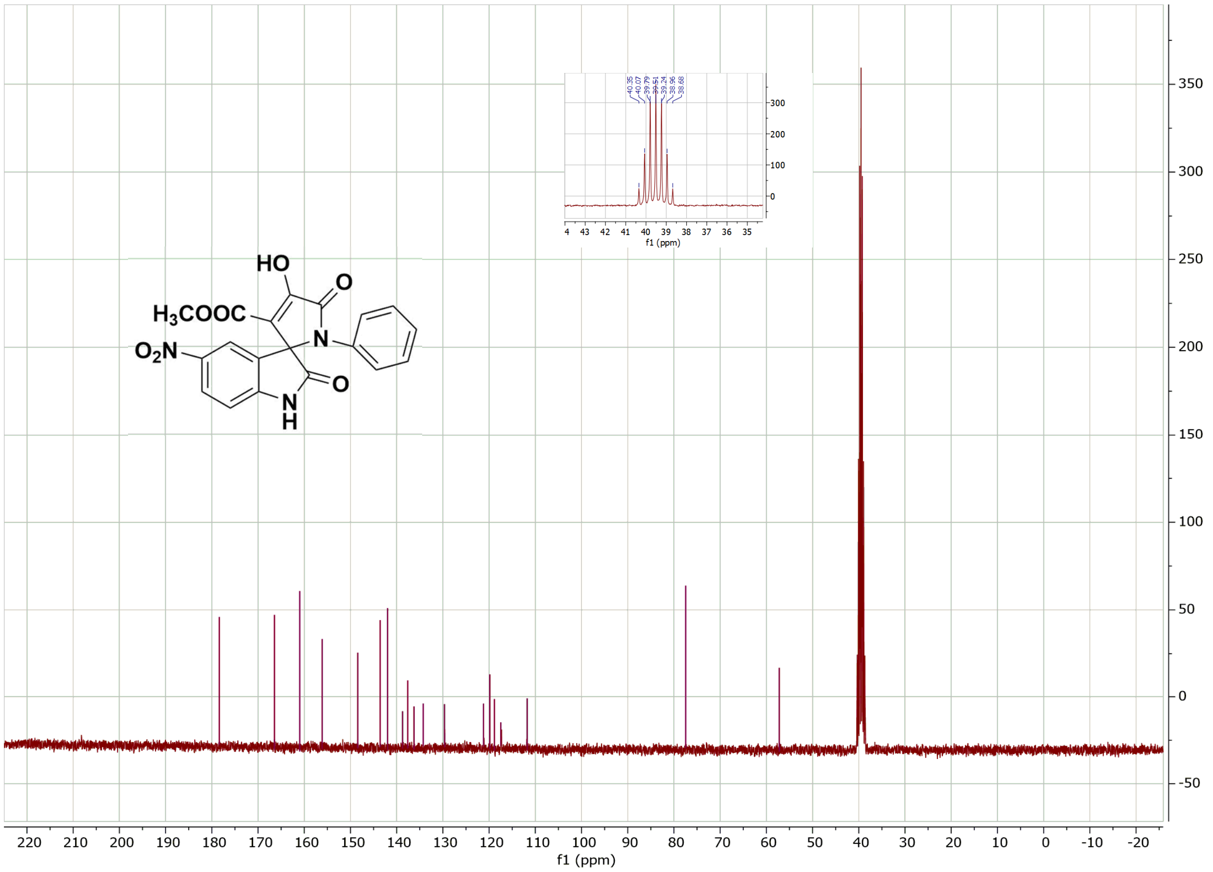

^{1}H NMR Spectra (4g)

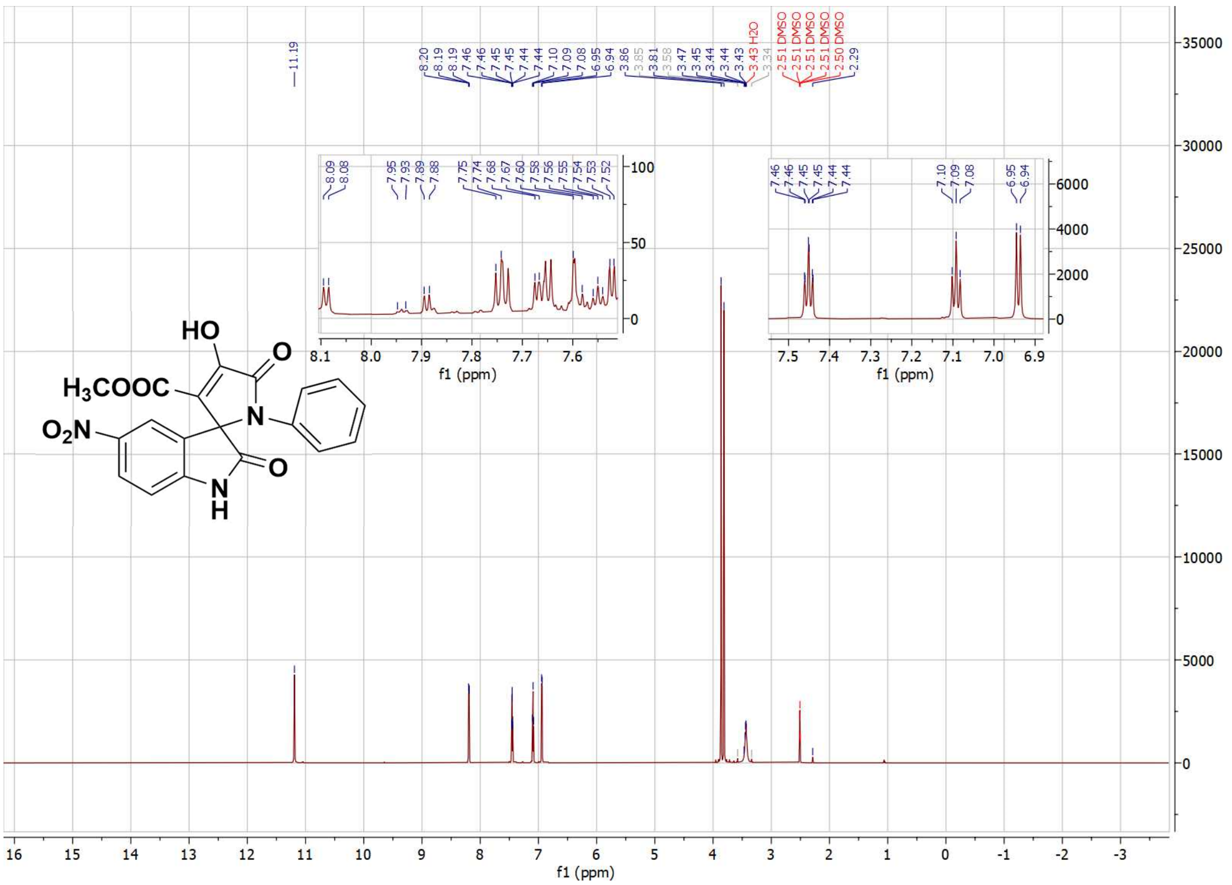

FT-IR Spectra (4h)

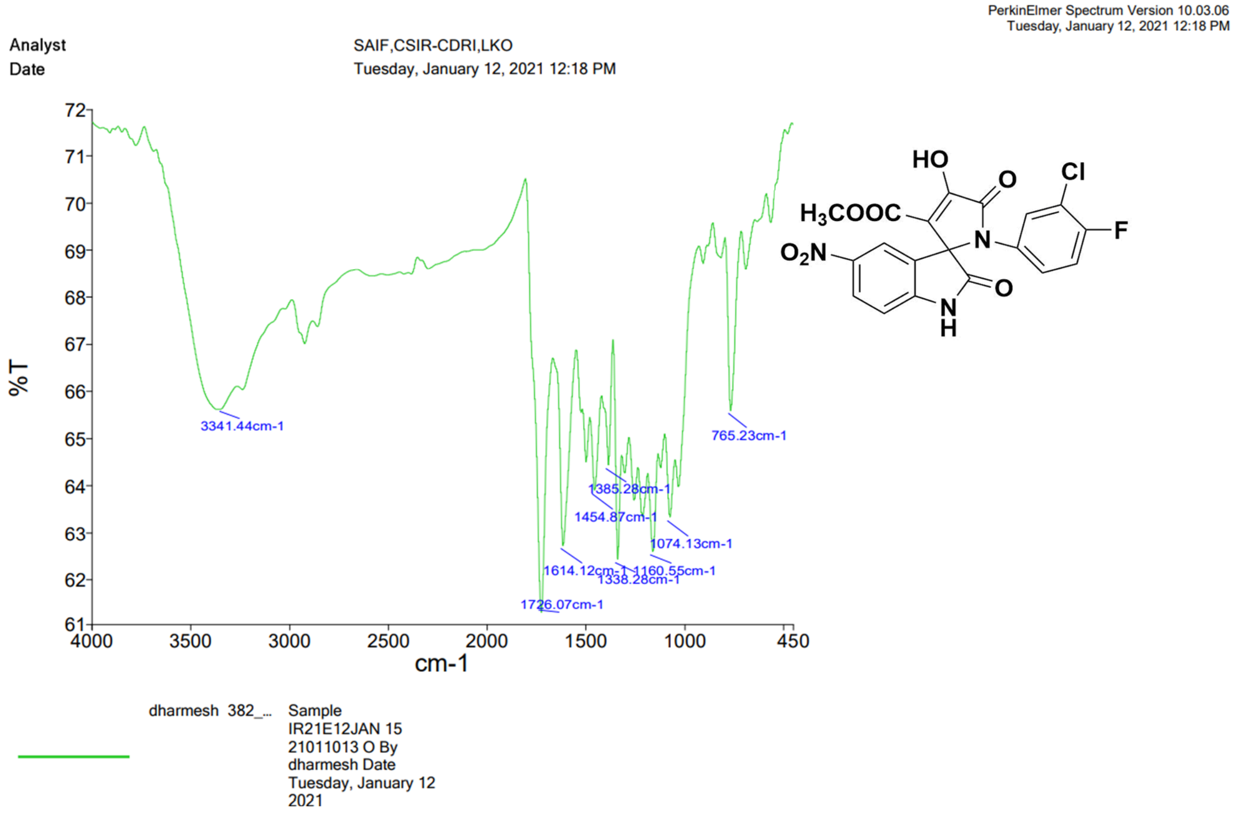

^{1}H NMR Spectra (4h)

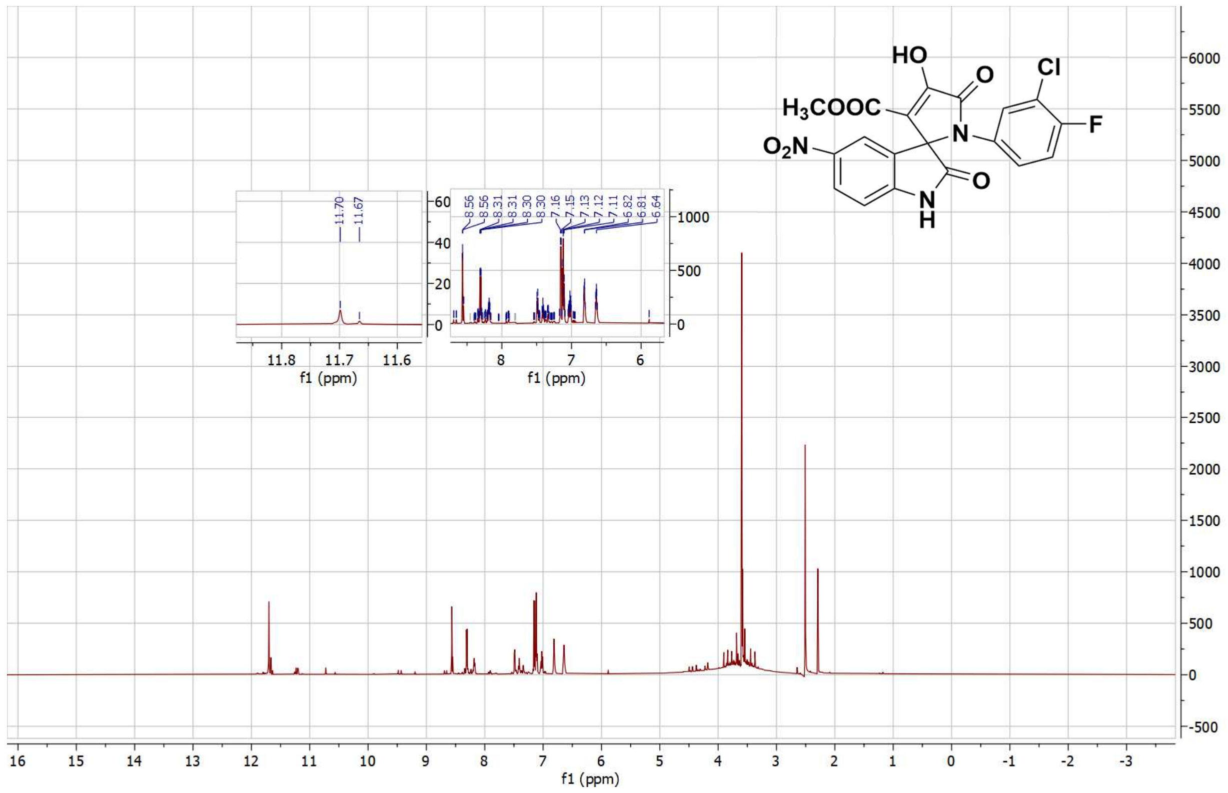

^{13}C NMR Spectra (4h)

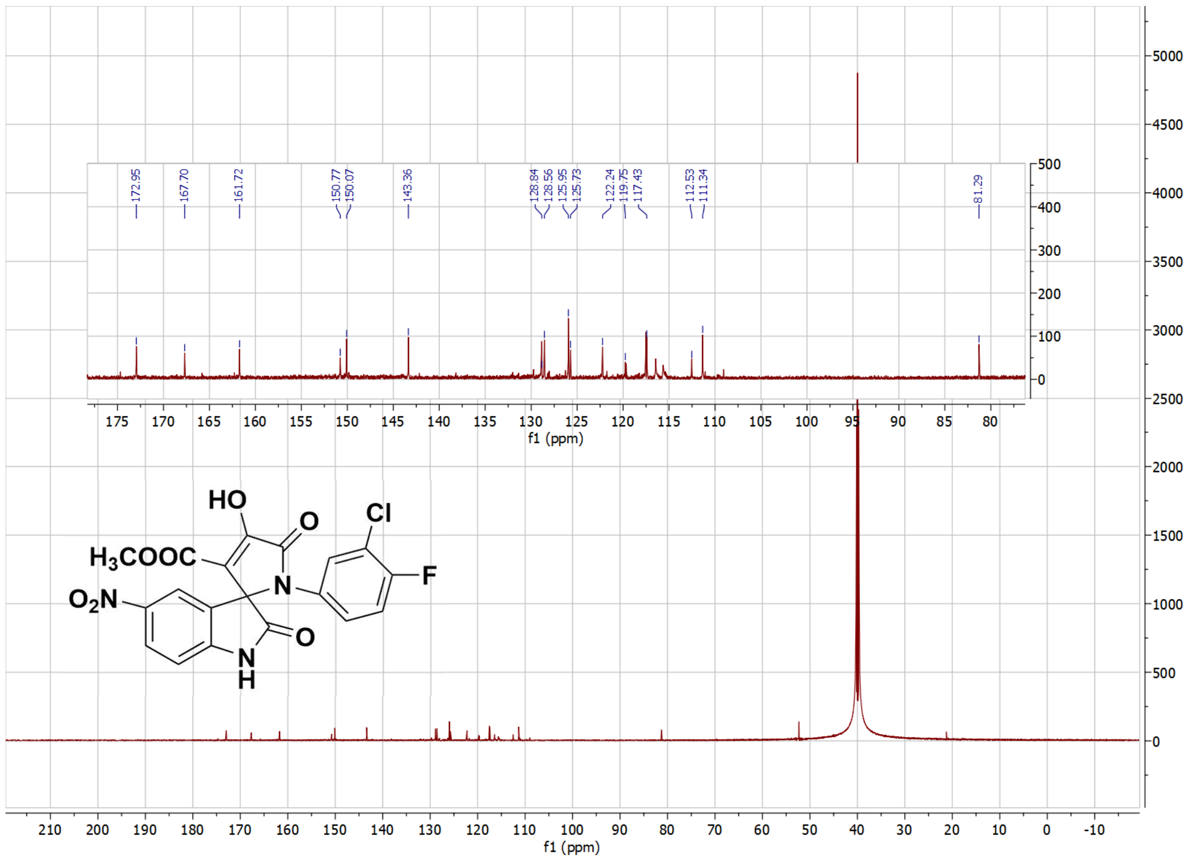

HRMS (4h)

Qualitative Analysis Report

**Data Filename**	TZ-8-O.d	**Sample Name**	TZ-8-O
**Sample Type**	Sample	**Position**	Vial 34
**Instrument Name**	Instrument 1	**User Name**	
**Acq Method**	Direct Mass-2017.m	**Acquired Time**	2/4/2021 10:32:28 PM
**IRM Calibration Status**	Some Ions Missed	**DA Method**	Default.m
**Comment**			

Sample Group **Info.**
Acquisition SW Version 6200 series TOF/6500 series Q-TOF B.05.00 (B5042.0)

User Chromatograms

Fragmentor Voltage 118 **Collision Energy** 0 **Ionization Mode** ESI

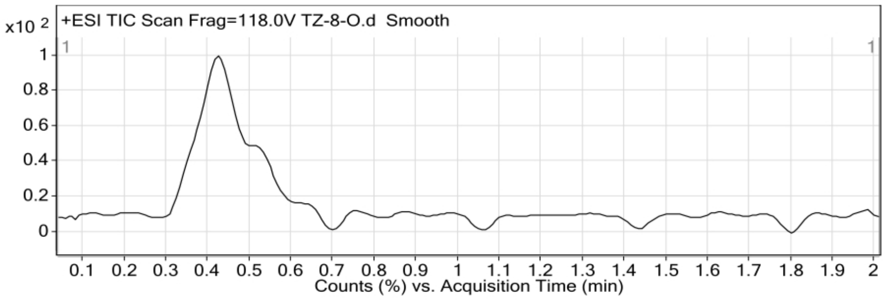

User Spectra

Fragmentor Voltage	Collision Energy	Ionization Mode
118	0	ESI

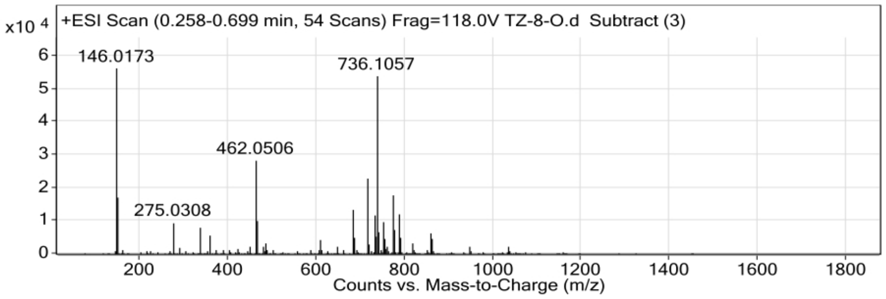

Peak List

m/z	z	Abund
146.0173	1	56403.63
148.0143	1	17375.24
462.0506	1	28396.44
682.0977	1	13583.08
714.1237	1	23047
736.1057	1	54071.12

737.109	1	17843.09
738.1043	1	20839.02
773.1971	1	17944.33
787.2125	1	12071.2

Compounds

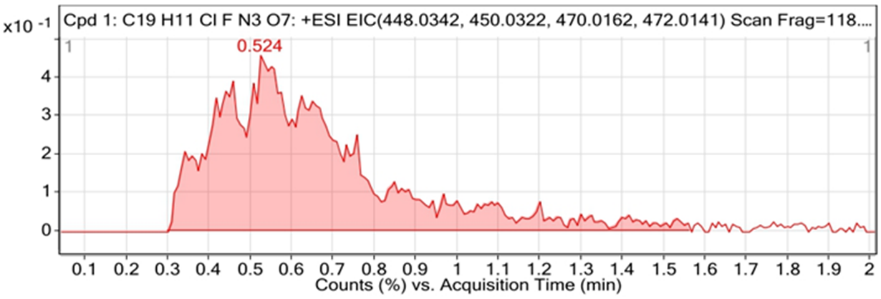

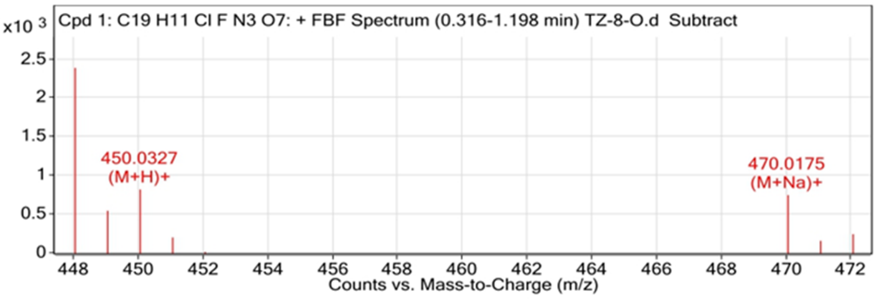

Peak List

m/z	z	Abund	Formula	Ion
448.0347	1	2399.17	C19H12ClFN3O7	(M+H)+
449.0382	1	559.81	C19H12ClFN3O7	(M+H)+
450.0327	1	834.82	C19H12ClFN3O7	(M+H)+
451.0342	1	211.27	C19H12ClFN3O7	(M+H)+
452.0451	1	23.47	C19H12ClFN3O7	(M+H)+
470.0175	1	762.7	C19H11ClFN3NaO7	(M+Na)+
471.0194	1	165.7	C19H11ClFN3NaO7	(M+Na)+
472.014	1	255.09	C19H11ClFN3NaO7	(M+Na)+

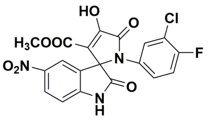

Chemical Formula: $C_{19}H_{11}ClFN_3O_7$
Molecular Weight: 448
HRMS (ESI-TOF m /z) (M + H) + calculated for $C_{19}H_{12}ClFN_3O_7^+$ 449.00784
Found 448.0347; 449.0382; 450.0327; 451.0342; 452.0451
HRMS (ESI-TOF m /z) (M + Na) + calculated for $C_{19}H_{11}ClFN_3NaO_7^+$ 470.28976
Found 470.0175; 471.0194; 472.014.

Supplementary Table S1:Drug-likeness studies of novel SOXs (4a-h).

Molecular descriptors	Novel spirooxindole-pyrrolines							
	**4a**	**4b**	**4c**	**4d**	**4e**	**4f**	**4g**	**4h**
MW	452.77	402.76	350.32	429.22	481.66	531.66	395.32	447.76
HBD	2	2	2	2	2	2	2	2
HBA	8	6	5	5	6	8	7	8
LogP	2.37	2.2	1.92	2.2	2. 48	2.91	1. 48	1.64
TPSA (Å^2)	95.94	95.94	95.94	95.94	95.94	95.94	141.76	141.76
Rotatable bonds (RB)	4	3	3	3	3	4	4	4
Lipinski's violations	0	0	0	0	0	1	0	0
Veber's violations	0	0	0	0	0	0	1	1

Supplementary Table S2:ADME indices of novel SOXs (4a-h).

S.N.	Compounds	Aq. Solubility (mg/L)	BBB (C. Brain/C. Blood	Caco2 cell permeability (nm/sec)	MDCK cell Permeability (nm/sec)	Skin permeability (logKp; cm/h)	PPB (%)	HIA (%)	CYT. p450 2D6 inhibition	CYT. p450 2D6 substrate
1.	**4a**	3.629	0.154	20.206	1.371	-2.810	92.007	94.745	Non	Non
2.	**4b**	7.049	0.105	20.858	3.232	-4.233	89.411	94.685	Non	Non
3.	**4c**	100.057	0.420	20.861	9.255	-4.023	91.875	94.290	Non	Non
4.	**4d**	9.505	0.623	19.630	0.896	-3.932	92.326	94.896	Non	Non
5.	**4e**	0.653	0.212	19.921	0.380	-4.167	91.158	95.430	Non	Non
6.	**4f**	0.330	0.364	19.869	0.155	-2.723	91.983	95.580	Non	Non
7.	**4g**	24.282	0.109	21.090	1.409	-4.032	90.939	81.852	Non	Non
8.	**4h**	1.685	0.013	19.275	0.458	-4.216	92.711	90.0635	Non	Non
9.	**Doxorubicin**	112.691	0.032	17.726	1.023	-4.698	32.789	31.9529	Non	Non

*Plasma protein binding (PPB); Blood brain barrier (BBB); Human intestinal absorption (HIA)
